# Vulnerability or Sensitivity to the Environment? Methodological Issues, Trends, and Recommendations in Gene–Environment Interactions Research in Human Behavior

**DOI:** 10.3389/fpsyt.2017.00106

**Published:** 2017-06-15

**Authors:** Caroline Leighton, Alberto Botto, Jaime R. Silva, Juan Pablo Jiménez, Patrick Luyten

**Affiliations:** ^1^Departamento de Psiquiatria y Salud Mental Oriente, Universidad de Chile, Santiago, Chile; ^2^Millennium Institute for Research in Depression and Personality – MIDAP, Ministry of Economy, Santiago, Chile; ^3^Centro de Apego y Regulación Emocional (CARE), Facultad de Psicología, Universidad del Desarrollo, Santiago, Chile; ^4^Clinica Alemana de Santiago, Santiago, Chile; ^5^Clinical Psychology, University of Leuven, Leuven, Belgium; ^6^Research Department of Clinical, Educational and Health Psychology, University College London, London, United Kingdom

**Keywords:** gene–environment interaction, diathesis-stress model, differential susceptibility model, life events, early adversity, psychopathology

## Abstract

Research on the potential role of gene–environment interactions (GxE) in explaining vulnerability to psychopathology in humans has witnessed a shift from a diathesis-stress perspective to differential susceptibility approaches. This paper critically reviews methodological issues and trends in this body of research. Databases were screened for studies of GxE in the prediction of personality traits, behavior, and mental health disorders in humans published between January 2002 and January 2015. In total, 315 papers were included. Results showed that 34 candidate genes have been included in GxE studies. Independent of the type of environment studied (early or recent life events, positive or negative environments), about 67–83% of studies have reported significant GxE interactions, which is consistent with a social susceptibility model. The percentage of positive results does not seem to differ depending on the gene studied, although publication bias might be involved. However, the number of positive findings differs depending on the population studied (i.e., young adults vs. older adults). Methodological considerations limit the ability to draw strong conclusions, particularly as almost 90% (*n* = 283/315) of published papers are based on samples from North America and Europe, and about 70% of published studies (219/315) are based on samples that were also used in other reports. At the same time, there are clear indications of methodological improvements over time, as is shown by a significant increase in longitudinal and experimental studies as well as in improved minimum genotyping. Recommendations for future research, such as minimum quality assessment of genes and environmental factors, specifying theoretical models guiding the study, and taking into account of cultural, ethnic, and lifetime perspectives, are formulated.

## Introduction

Every human being is unique, despite sharing over 99% of genetic material with the rest of the human species (1, 2). Recent theoretical models stress the fact that a person’s relationship with his/her environment from the moment of conception can be assumed to play a crucial role in this uniqueness (3–5). The answer of what makes us distinctively different from other human beings may lie in the continuous reciprocal interaction between the environment and our genome. Such gene–environment relations are thought to result from both gene–environment correlations (rGE) and gene–environment interactions (GxE).

Research on rGE explores the role of genes in the exposure to environmental factors (6, 7). rGE refers to the tendency of individuals to select and generate their environment based on genetic features that influence behavior, thoughts, and feelings. Three types of rGE have been described in the literature: (a) passive, (b) reactive, provocative, or evocative, and (c) active or selective (8). (a) Passive rGE refers to the situation in which children inherit from their parents not only a genetic constitution but also the environment in which they are raised (6) (e.g., they inherit intellectual curiosity). The association between genetically related individuals is a requirement for passive rGE. (b) Evocative, provocative, or reactive rGE refers to the tendency of certain genetically influenced behaviors or temperamental features to elicit certain types of responses from people within their environment (e.g., a child with a difficult temperament is more likely to elicit negative parenting behaviors). (c) Active or selective rGE refers to the active generation of certain environments based on genetic tendencies. This refers to the association between genetic features of the individual and the environmental niches that the individual selects or generates (e.g., a child with intellectual curiosity will tend to find intellectually rich environments, while a child with behavioral disorder will seek peers with similar behaviors; that is, people who are more extroverted may seek very different social environments from those who are shy and withdrawn) (6).

Gene–environment interactions, on the other hand, explain why people respond differently to environmental factors (e.g., why certain individuals are more prone to depression after being exposed to negative life events) (9). Until relatively recently, GxE were thought to be rare in psychiatry, but research over the past decades has shifted toward a focus on GxE (10, 11).

Gene–environment correlations and GxE are not mutually exclusive. A polymorphism may correlate with some traits that generate changes in the environment. An example of such a mediational model is the finding that the short allele of the polymorphism of the promoter region linked to the serotonin transporter gene (5HTTLPR) has been shown to correlate with neuroticism (12, 13), which in turn has been shown to be related to a tendency to have a negative interpretation bias related to life events (14). Moderator models in this context imply that there is an interaction with environmental factors. For example, studies suggest that 5HTTLPR polymorphism may interact with negative life events in the prediction of depression, but also with social support, leading to lower levels of depression (15–17).

There is now increasing consensus that most common psychiatric disorders, such as depression and anxiety, are best explained as complex disorders involving dysfunctions in several biological systems in interaction with environmental factors. One of the earliest studies of GxE was reported by Kendler and colleagues (9). This study overthrew the concept of reactive or endogenous depression, because those individuals with a greater genetic risk for depression were shown to be also more reactive to negative environmental events. In 2003, Caspi and colleagues (18) published a ground-breaking study that reported that carrying the short allele of the 5HTTLPR polymorphism interacted with both early and recent negative events to predict depression. Yet, findings have not always been consistent. Two meta-analyses, for instance, failed to corroborate an interaction between the 5HTTLPR polymorphism and stressful life events in predicting depression (19, 20). By contrast, a meta-analysis by Uher and McGuffin (21) did find evidence for an interaction between the 5HTTLPR polymorphism and adversity in predicting depression. Differences between these studies’ conclusions may be due to differences in their methodology and inclusion criteria. But it is clear that there still is controversy regarding the role of GxE and rGE in psychiatric disorders (17, 22–25).

Importantly, until recently, research in this area has mainly focused on studying the moderation of *negative* environments from a diathesis-stress perspective. In recent years, studies have begun to measure potential GxE and rGE with regard to *positive* events and experiences (17, 26–29). This shift has led to the formulation of what has been variously called *differential susceptibility, biological sensitivity to context*, or *social susceptibility genes* models (30–37). These models contend that individuals have differences in developmental plasticity and, more generally, susceptibility to environmental influences, with some individuals being more affected than others by both negative and positive contextual conditions. Those allegedly “vulnerable” individuals, who are most adversely affected by different stressors, may thus be the very same individuals who reap the most benefit from environmental support and enrichment, including the absence of adversity (31).

Cultural factors may also come into play here. For instance, some GxE seem quite robust in Western cultures but have not been replicated in Eastern cultures (38), an issue to which we return in detail below. Further, GxE may also differ along the course of development, with some interactions observed at some points during development but not during other developmental stages (39), and some may be gender dependent.

The aim of this paper is therefore to critically review the research on GxE with the aim of fostering research in this area. Specifically, we provide a systematic qualitative review of research on all genes that have been investigated in GxE research, focusing on five areas: (a) the candidate genes studied; (b) the phenotype or effect studied for each gene; (c) the type of environment investigated; (d) the samples investigated in terms of age group and geographical regions where the studies took place; and (e) the methodological considerations. Based on this review, we also formulate a number of recommendations for future research.

## Methods

For this review, empirical studies published in English in peer-reviewed journals, from January 2002 (the year Caspi’s seminal study was published (40)) to January 14, 2015, were retrieved using several search engines (PubMed, PsycINFO, and Google Scholar) and the following combinations of subject headings: Affiliative and polymorphism, affiliative and gene, prosocial and polymorphism, prosocial and gene, social behavior and polymorphism, social behavior and gene, susceptibility polymorphism, plastic polymorphism, GxE and social behavior, GxE and polymorphism, gene and environment interaction; and a second search using the individual gene (e.g., 5HTT), interaction and stress, emotion, or trauma, and depression. Finally, references of retrieved papers were hand searched.

Inclusion criteria were

(a) Studies had to investigate the interaction between gene polymorphisms and environment in the prediction of personality traits, behavior, or mental health disorders in humans. We included studies that focused on GxE in explaining behavioral and mental health outcomes (i.e., depression), as well as studies on the neurobiological mechanisms involved, as these studies may contribute to our understanding of the underlying neural circuits.
(b) The environmental factor could be a naturally occurring event (e.g., a disaster) or experimentally manipulated and measured prospectively or retrospectively by using observational measures, interviews, or questionnaires. Studies that included only proxies of environmental factors [e.g., maternal smoking, alcohol use during pregnancy, (41), or peripartum (42)] were excluded. Similarly, studies that included exposure to substances [e.g., drugs, alcohol, and oxytocin (43–45)] were excluded. Similarly, studies using a measure of “perceived” stress were excluded, because perceived stress could be confounded with G or reflect GxG effects. Genotyping criteria (success rate and reported Hardy–Weinberg equilibrium; HWE) were extracted from all papers, but these data were not used as an exclusion criterion.

**Figure 1:**
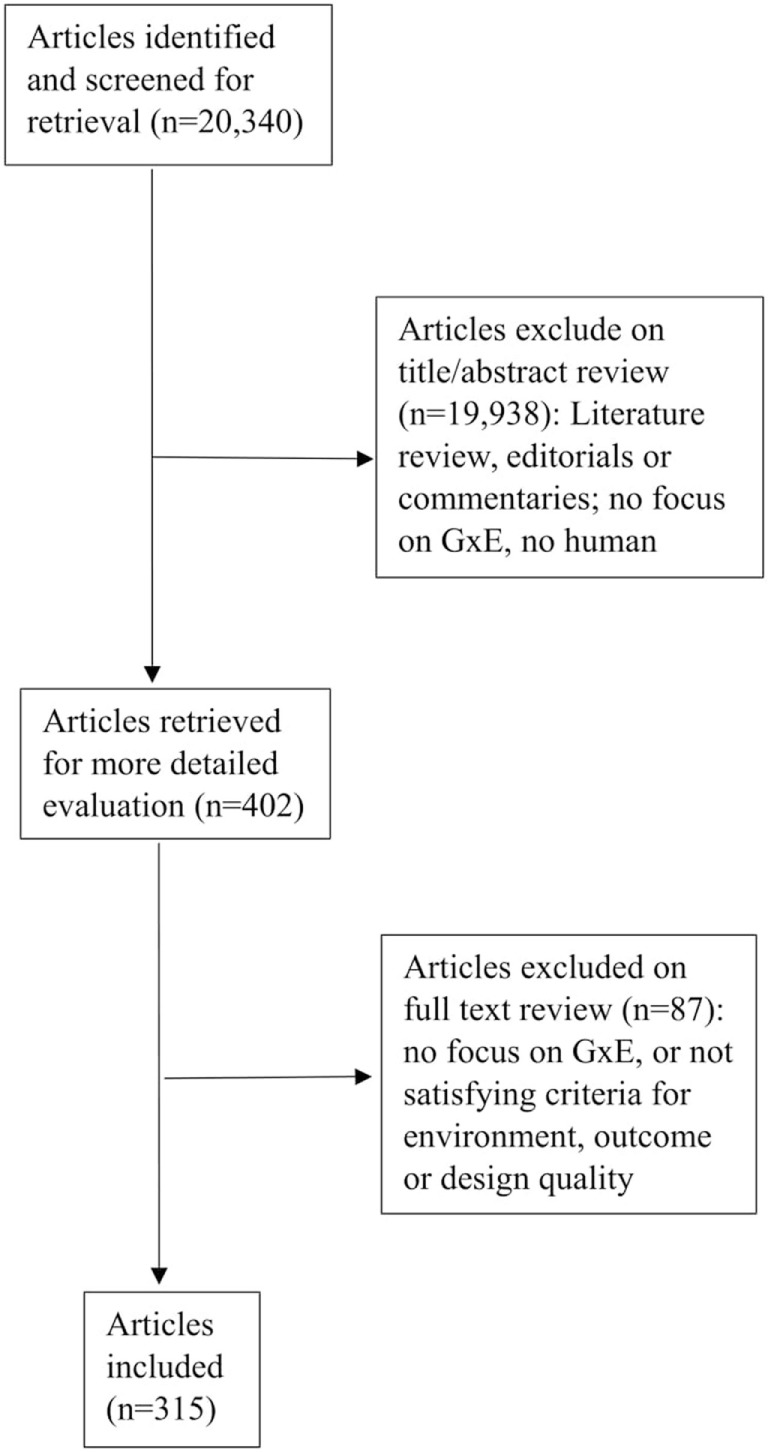
Flowchart showing the search and selection of articles in this study.

Two of the authors (Caroline Leighton and Alberto Botto) reviewed all titles and abstracts of the 20,340 retrieved articles. Figure 1 details the flowchart of the papers. This resulted in 315 papers, pertaining to 160 original samples. A detailed list of the studies can be obtained from the first author.

Data were then extracted pertaining to five topics: (a) the candidate genes studied; (b) the outcome or effect studied for each gene [the effects of the GxE were classified as focusing on: (1) psychopathology, depression, or other, (2) social behavior, (3) social cognition, (4) stress regulation, (5) attachment, (6) personality traits, (7) neurobiology]; (c) the type of environmental factor investigated; (d) the age and geographical regions (or country) where the studies took place; and (e) the methodological considerations or quality of the studies identified.

Given the variety in designs, outcomes, and environment measures used in these studies, a quantitative meta-analysis was not indicated, and therefore, a qualitative review is provided.

## Results

### Candidate Genes

The number of publications of GxE since Caspi’s seminal study in 2002 (40) clearly demonstrates the exponential growth of studies in this area. In total, we identified polymorphisms of 34 different genes that have been studied in GxE research (see Table 1) in 315 papers using 160 original samples (see below).

**Table 1:** Type and number of genes included in gene–environment interactions studies.

Gene	Name	No. of articles
SLC6A4 (5HTT)	Serotonin transporter	162
BDNF	Brain-derived neurotrophic factor	44
DRD4	Dopamine receptor	36
MAOA	Monoamine oxidase A	36
OXTR	Oxytocin receptor	19
COMT	Catechol-*O*-methyltransferase	17
5HTR (1A/1B/2A/2C/3A)	Serotonin receptors	15
DRD2	Dopamine receptor	13
FKBP5	FK506 binding protein 5	10
CRHR1	Corticotropin-releasing hormone receptor 1	9
SLC6A3 (DAT1)	Dopamine transporter	6
TPH1/TPH2	Tryptophan hydroxylase	5
NR3C1 (GR)	Glucocorticoid receptor	4
NR3C2 (MR)	Mineralocorticoid receptor	4
OPRM1	μ1 Opioid receptor	3
GABRA2/GABRG1	γ1 and α2 subunits of GABA-A receptor	3
RGS2	Regulator of G-protein signaling 2	3
CHRM2	Cholinergic muscarine 2 receptor	2
ANKK1	Ankyrin repeat and kinase domain containing 1	2
PER1/PER2	Period circadian clock 1 and 2	2
OXT	Oxytocin	2
NPY	Neuropeptide Y	1
ACE	Angiotensin 1 converting enzyme	1
GRIN2B	Glutamate receptor, ionotropic, NMDA 2B	1
NPSR1	Neuropeptide S receptor	1
CACNA1C	Calcium channel, voltage-dependent, L type, α 1C subunit	1
CREB1	cAMP responsive element binding protein 1	1
FOXP2	Forkhead box protein 2	1
GALR1/GALR2/GALR3	Galanin receptors	1
MAOB	Monoamine oxidase B	1
SLC6A2 (NET)	Norepinephrine transporter	1
NOS1	Nitric oxide synthase 1 (neuronal)	1
ODC1	Ornithine decarboxylase 1	1
DRD1/DRD3/DRD5	Dopamine receptor	1

The most investigated gene is 5HTT (SLC6A4), with about half (51.4%, 162 articles) of the total number of studies on GxE focusing on the 5HTTLPR polymorphism, followed by the brain-derived neurotrophic factor gene (BDNF: *n* = 44/315 studies, 13.9%), dopamine receptor D4 gene (DRD4: *n* = 36/315 studies, 11.4%), monoamine oxidase A gene (MAOA: *n* = 36/315 studies, 11.4%), oxytocin receptor gene (OXTR: *n* = 19/315 studies, 6%), catechol-*O*-methyltransferase gene (COMT: *n* = 17/315 studies, 5.4%), serotonin receptor genes (i.e., 5HTR 1A/1B/2A/2C/3A: *n* = 15/315 studies, 4.7%), dopamine receptor D2 gene (DRD2: *n* = 13/315 studies, 4.1%), corticotropin-releasing hormone receptor 1 gene (CRHR1: *n* = 9/315 studies, 3.8%), and FK506 binding protein gene (FKBP5: *n* = 10/315 studies, 3.2%).

### Outcome Studied

**Figure 2:**
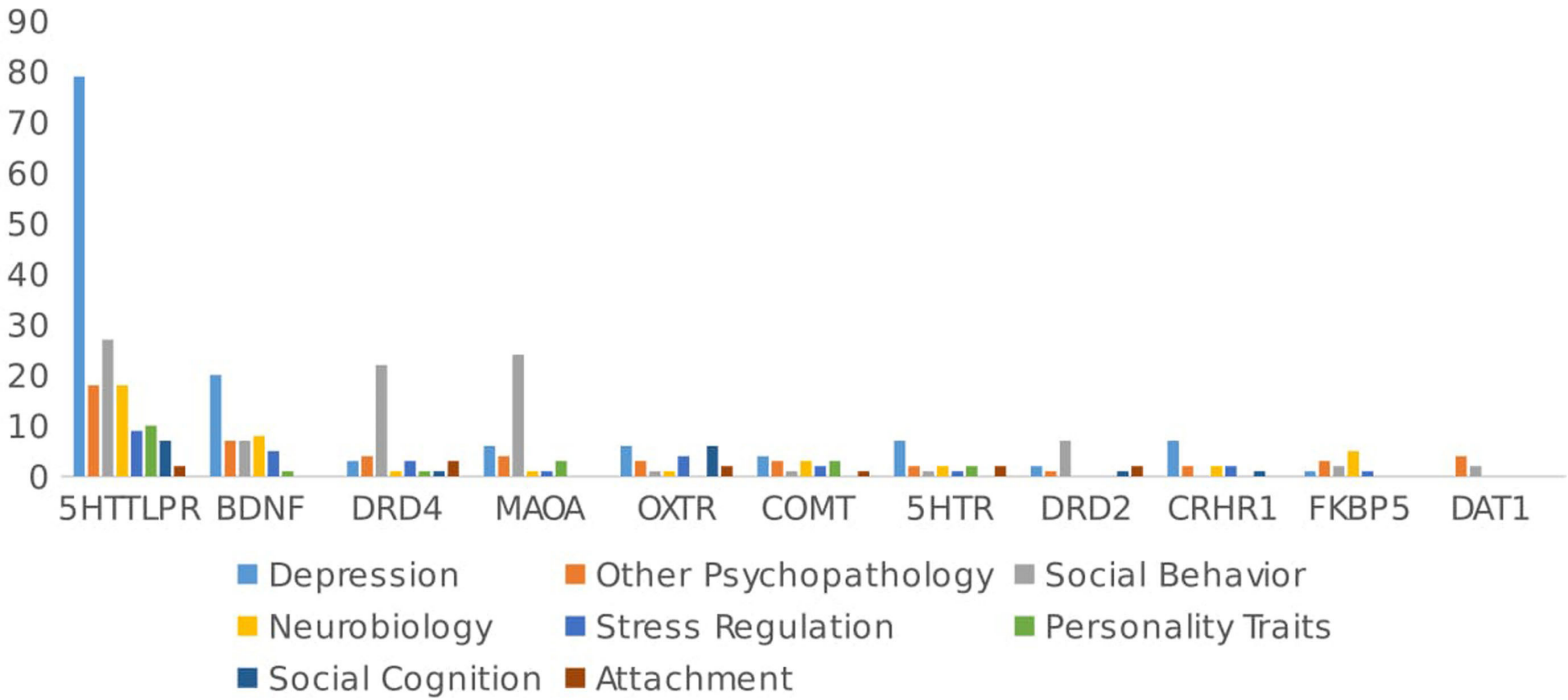
Outcome studied by gene.

Figure 2 summarizes the outcome studied by gene. Almost half of the studies focused on different types of psychopathology (*n* = 150/315 studies, 46.8% of the total number of papers). Depression has been by far the most studied pathology (*n* = 102/315 papers, 32.3%), with studies focusing mainly on 5HTTLPR (*n* = 79/102, 77.5%) and BDNF (*n* = 20/102, 19.6%), and the remainder investigating 5HTR (1A/1B/2A/2C/3A) (*n* = 7/102, 6.9%), CRHR1 (*n* = 7/102, 6.9%), MAOA (*n* = 6/102, 5.9%), and OXTR (*n* = 6/102 papers, 5.9%). These genes have been mostly studied in interaction with early stressful events or chronic stress to predict depression and (less frequently) anxiety.

By contrast, Figure 2 shows that social behavior (*n* = 86/315, 21.3% of total papers) has been primarily studied in interaction with genes related to the dopaminergic system (DRD4, DRD2, MAOA, DAT1). These genes have been mostly studied in interaction with parenting to predict behaviors such as criminal activity, alcohol use, and behavioral problems in adolescents.

Studies on the neurobiological mechanisms (studies that include as an outcome intermediate pathways that could be involved in the GxE mechanism, i.e., changes in cortisol levels or changes in methylation rates) involved in GxE have been relatively scarce, at least in humans (*n* = 39/315, 12.2% of the total number of papers). Genes related to the glucocorticoid system have focused the most on neurobiological outcomes (e.g., FKBP5, GR, and CRHR1), and only a small proportion of articles on 5HTTLPR (*n* = 18/162) and BDNF (*n* = 8/44) have focused on the neurobiological outcomes of GxE.

Stress regulation was studied in 6.6% (*n* = 21/315) articles of GxE, with most studies focused on corticoid-related genes (CRHR1 *n* = 2/12, 16.7%, GR *n* = 1/4, 25% and FKBP51/10, 10%) and the remainder on BDNF, OXTR, and DRD4.

Personality traits were included as an effect of GxE studies in only 6.6% of the papers (*n* = 21/315), with studies focusing mainly on 5HTTLPR [10/162 papers (6.2%), including impulsivity, neuroticism, emotional dysregulation, and self-esteem], DRD4 (*n* = 1, impulsivity), BDNF (*n* = 1), NPSR (*n* = 1, anxiety sensitivity), TPH1/TPH2 (*n* = 2, harm avoidance and impulsivity), COMT (3/17 impulsivity), and 5HTR (*n* = 2).

Social cognition as an effect of GxE was studied in only 4.3% (*n* = 14/315) of the articles, mostly focusing on the oxytonergic system genes (OXT and OXTR).

Attachment was included in only 2.2% (*n* = 7/315) of the articles, with studies focusing on DRD4 (*n* = 3), DRD2 (*n* = 2), 5HTR (*n* = 2), 5HTTLPR (*n* = 2/162), OPRM1 (*n* = 1), MR/NR3C2 (*n* = 1), OXTR (*n* = 2), and COMT (*n* = 1).

As Figure 2 shows, analyzing papers by genes, again, most articles have focused on the 5HTTLPR polymorphism in relation to psychopathology (*n* = 97/162, 59.9%), especially depression (*n* = 79, 48.9%). Further, three polymorphisms in the 5HTT gene have been implicated in treatment response and neuropsychiatric disorders. A 44 base pair (bp) ins/del polymorphism in the promoter region (5HTTLPR) produces primarily long and/or short alleles due to either 14 (short) or 16 (long) repeats of variably conserved 20–23 bp units. In addition, a 17–18 bp variable number tandem repeat (VNTR) found in intron 2 (StIn2) is expressed as triallelic content with 9, 10, or 12 repeats (StIn2.9, StIn2.10, or StIn2.12). Finally, a single nucleotide polymorphism (SNP), rs25531 A/G, located within the promoter polymorphic-linked region, alters the function of the long promoter allele.

The DRD4 gene has mainly been studied in social behavior phenotypes (*n* = 22, 61.1%). The gene has a polymorphism of a 48 bp VNTR in exon 3, ranging from 2 to 11 repeats. The 7-repeat version has been linked to greater sensitivity to the environment. The -521 C/T SNP located within the promoter has also been studied in GxE studies.

In the oxytonergic system, the polymorphism most studied for interactions with the environment is the OXTR gene, with studies focusing equally frequently on social cognition (*n* = 6, 32%) and depression (*n* = 6, 32%). The polymorphisms studied have been rs53576 (11 studies), rs237885 (2 studies), rs2254298 (4 studies), and rs53577 (2 studies). One study included linkage disequilibrium, calculating data using Haploview and the Center de’Etude du Polymorphism Humain data from the hapmap project (http://hapmap.ncbi.nlm.nih.gov/), including other 15 SNPs (46); these studies represent, however, only a selection of potential polymorphisms in the oxytonergic system.

### Type of Environment Investigated

**Figure 3:**
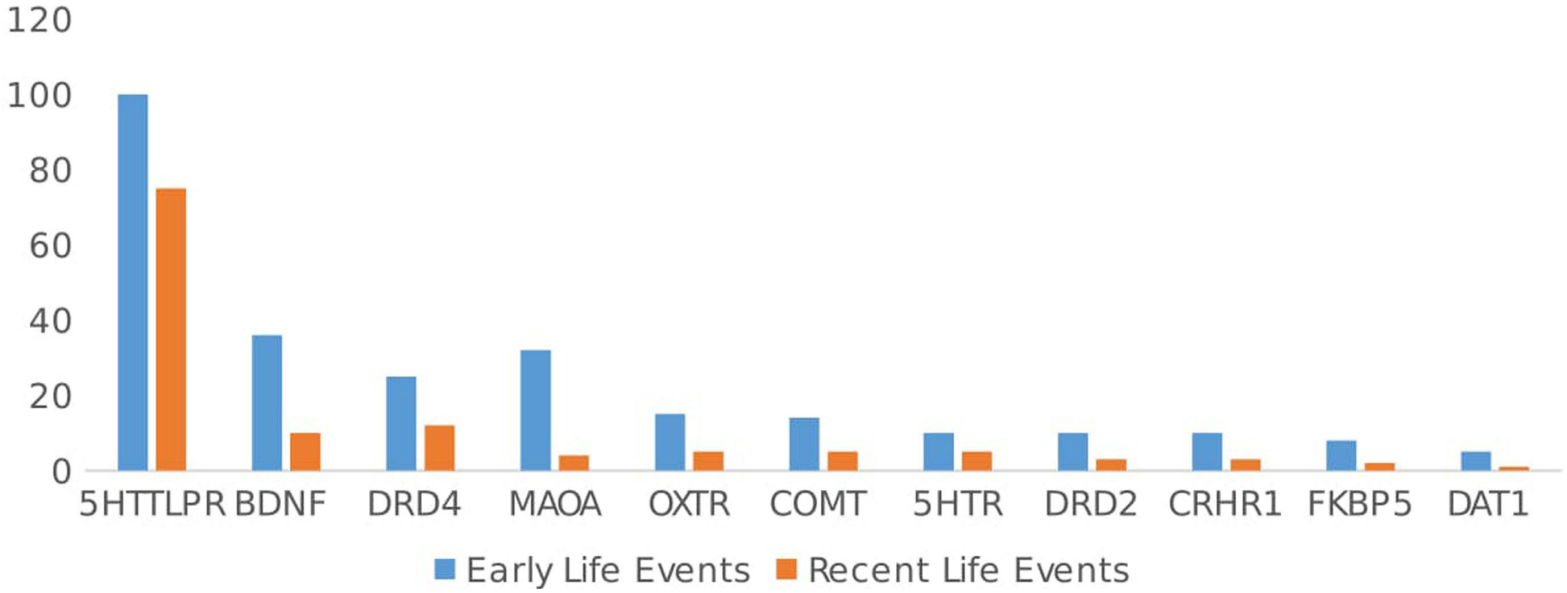
Type of environment studied (early vs. recent life events) by gene.

Among the kinds of environmental factors that have been studied, early and negative environments such as poor parenting and childhood trauma have been the most frequent focus of research. In total, 70.8% (*n* = 223/315) of the articles included early-life events (ELE). Recent-life events (RLE) such as psychosocial interventions, experimentally induced stress, and recent important experiences have been studied less often (*n* = 113/315, 35.9% of articles). Studies including ELE yielded evidence for GxE in 77% (172/223) of papers, and studies including RLE reported evidence for GxE in 73.5% (83/113) of papers; thus, approximately equal percentages of articles reported evidence for GxE compared with studies that reported negative results, χ^2^(1, *n* = 336) = 0.38, ns (see Figure 3).

**Figure 4:**
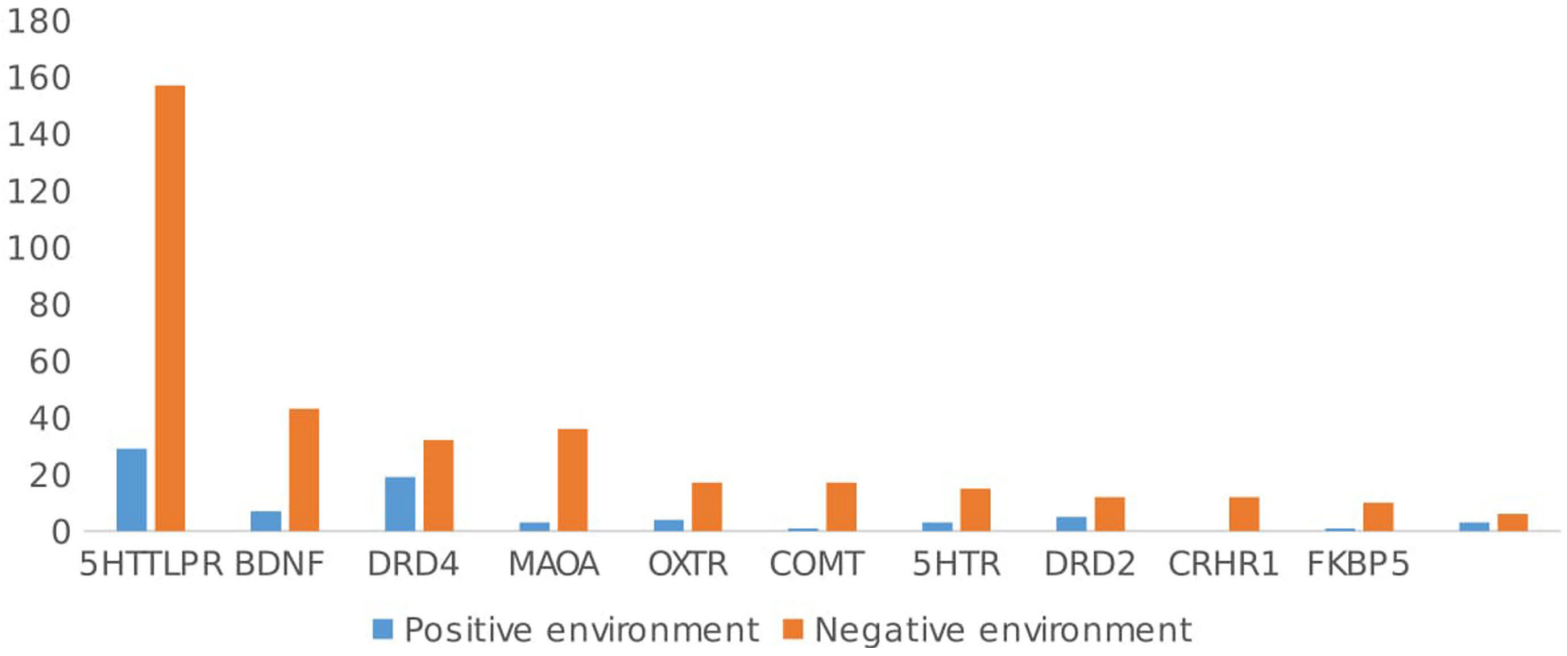
Type of environment studied (*negative* vs. *positive*) by gene.

While 95.9% (*n* = 302 articles) of the 315 articles included a negative environment, only 22.2% (*n* = 70/315 articles) focused on interactions with positive events (see Figure 4). In general, articles including a negative environmental factor found evidence for GxE in 78.1% (*n* = 236/302), while articles including a positive environmental factor found GxE in 81% (*n* = 57/70) of studies. Again, there was no significant difference in the relation between GxE and type of environment, χ^2^(1, *n* = 72) = 0.37, ns. Negative and positive environments showed the same evidence of GxE. Of the 57 articles including a positive environment, 29 studied the interaction from the perspective of the social susceptibility or differential sensitivity model. That is, the interaction between the same polymorphism and both positive and negative environments was simultaneously investigated. Among these studies, 24/29 (82.6%) showed an interaction between positive events and a genetic polymorphism. In general, independent of the type of environment studied (early or RLE, positive or negative environment), the proportion of papers that showed evidence for GxE was the same, χ^2^ (3, *n* = 708) = 1.76, ns.

Looking at the different polymorphisms separately, for 5HTTLPR, 62.3% (*n* = 101/162 studies) of the studies included ELE, while 45.7% (*n* = 74 studies) included RLE. Negative events were focused upon in 96.9% (*n* = 157/162) of the studies, and positive events were the focus in 17.9% (*n* = 29/162) of the studies. There were no significant differences in type of environment (positive, negative, ELE, or RLE) and evidence of GxE in 5HTTLPR studies, χ^2^(3, *n* = 362) = 0.09, ns.

For the DRD4, ELE were the focus of most articles (69.4% ELE vs. 33.3% RLE), but these studies focused much more on interactions with positive events than the 5HTTLPR studies did [52.8% of DRD4 articles included positive environment, vs. 17.9% of 5HTTLPR articles; χ^2^(3, *n* = 449) = 14.76, *p* < 0.05]. There was no difference in GxE reported and type of environment included (positive, negative, ELE, or RLE), χ^2^(3, *n* = 88) = 0.04, ns.

For the OXTR gene, ELE were studied more often than RLE 78.9% (*n* = 15/19) vs. 26.3% (*n* = 5/19), and the majority of articles (89.5%, *n* = 17/19) focused on negative environmental factors (vs. 21%, *n* = 4/19 on positive environments). Again, there was no difference in the type of environment studied (positive, negative, ELE, or RLE) and evidence found for GxE for OXTR, χ^2^(3, *n* = 41) = 0.08, ns.

### Age and Geographical Location of the Samples Included in the Studies

**Figure 5:**
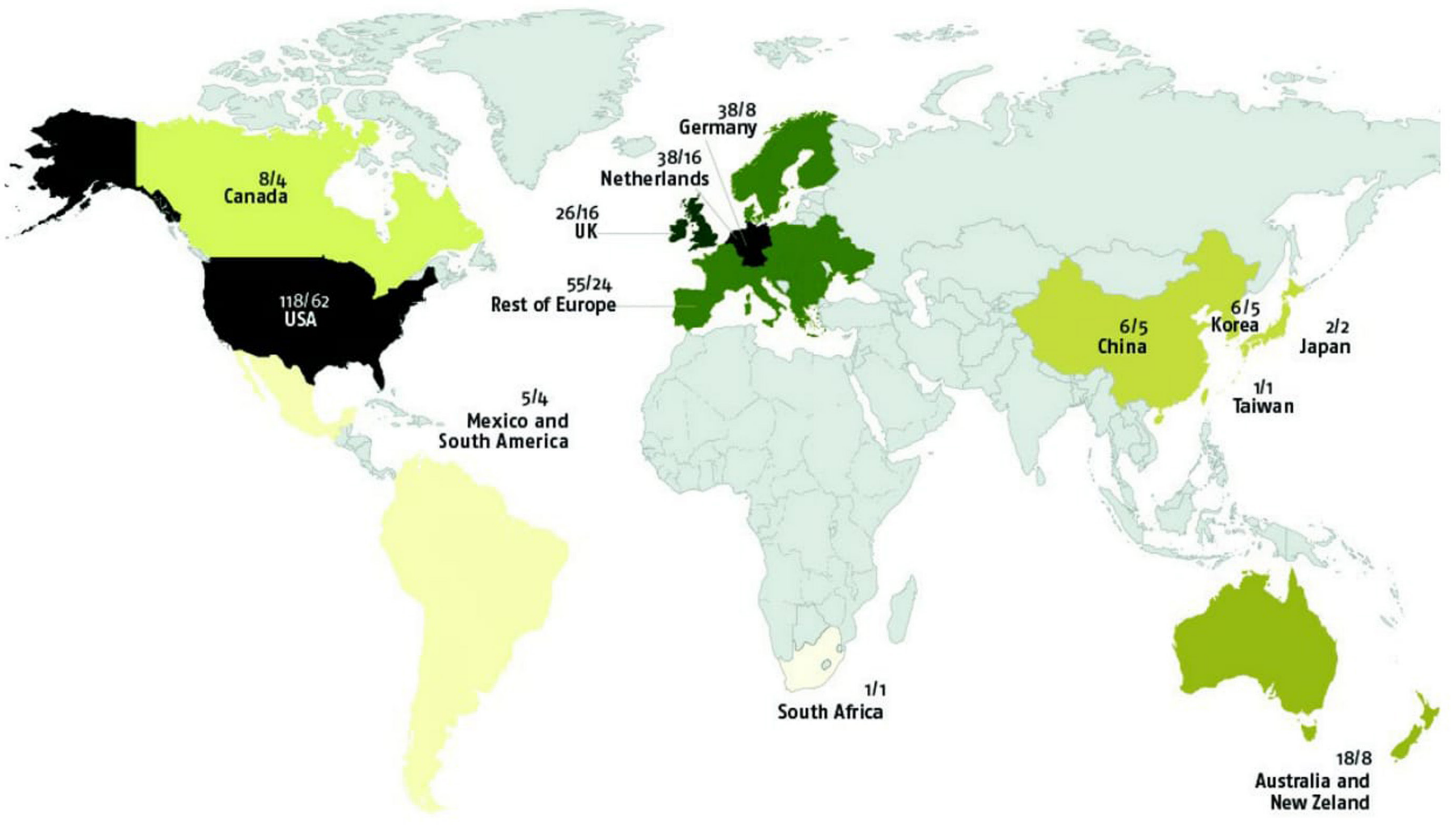
World distribution of gene–environment interaction studies (samples and original samples).

Figure 5 clearly shows that the vast majority of studies (almost 90%, *n* = 283/315) were conducted in North America or Europe. GxE were found in 80% (*n* = 241/301) of articles including Western samples and in 60% (*n* = 9/15) of articles samples from Eastern countries; this difference was not significant, χ^2^(1, *n* = 316) = 3.48, ns.

There were no differences between the country, continent, or ethnicity from which the sample came and evidence of GxE [χ^2^(9, *n* = 322) = 15.89, ns; χ^2^(5, *n* = 304) = 10.00, ns, and χ^2^(3, *n* = 237) = 3.94, ns, respectively].

The overlap of samples used in different research papers was very high. Of the 315 articles included in this review, only 96 used samples that did not overlap with samples reported on in other papers. Hence, 69.5% (*n* = 219) of the papers used samples that were also used in other GxE studies. From the 219 overlapping papers, original samples reduced to 64, so there were only 160 original samples studied for GxE. When taking into account overlap of samples in papers from different countries, the original proportion of 90% of the samples (articles) coming from the US or Europe diminished to 84.3%. Figure 5 shows the world distribution of the samples and original samples (not overlapped) used to study GxE.

**Figure 6:**
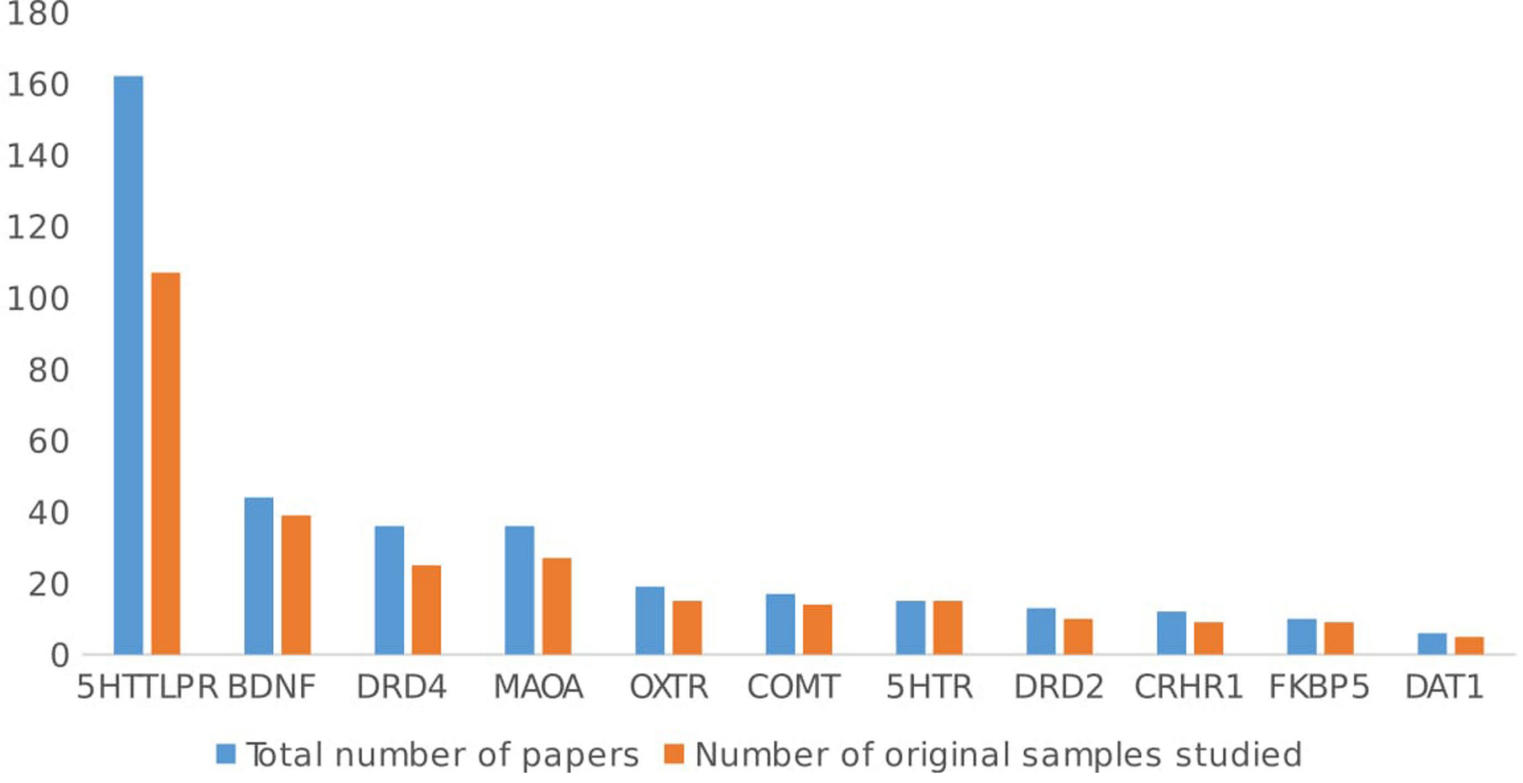
Overlap between study samples by gene.

Figure 6 shows the overlap by gene. 5HTT studies show the greatest overlap of samples (162 papers, using 102 samples), but there is no difference in the distribution of genes over the overlapped or not overlapped samples [χ^2^(10, *n* = 645) = 2.61, ns].

**Figure 7:**
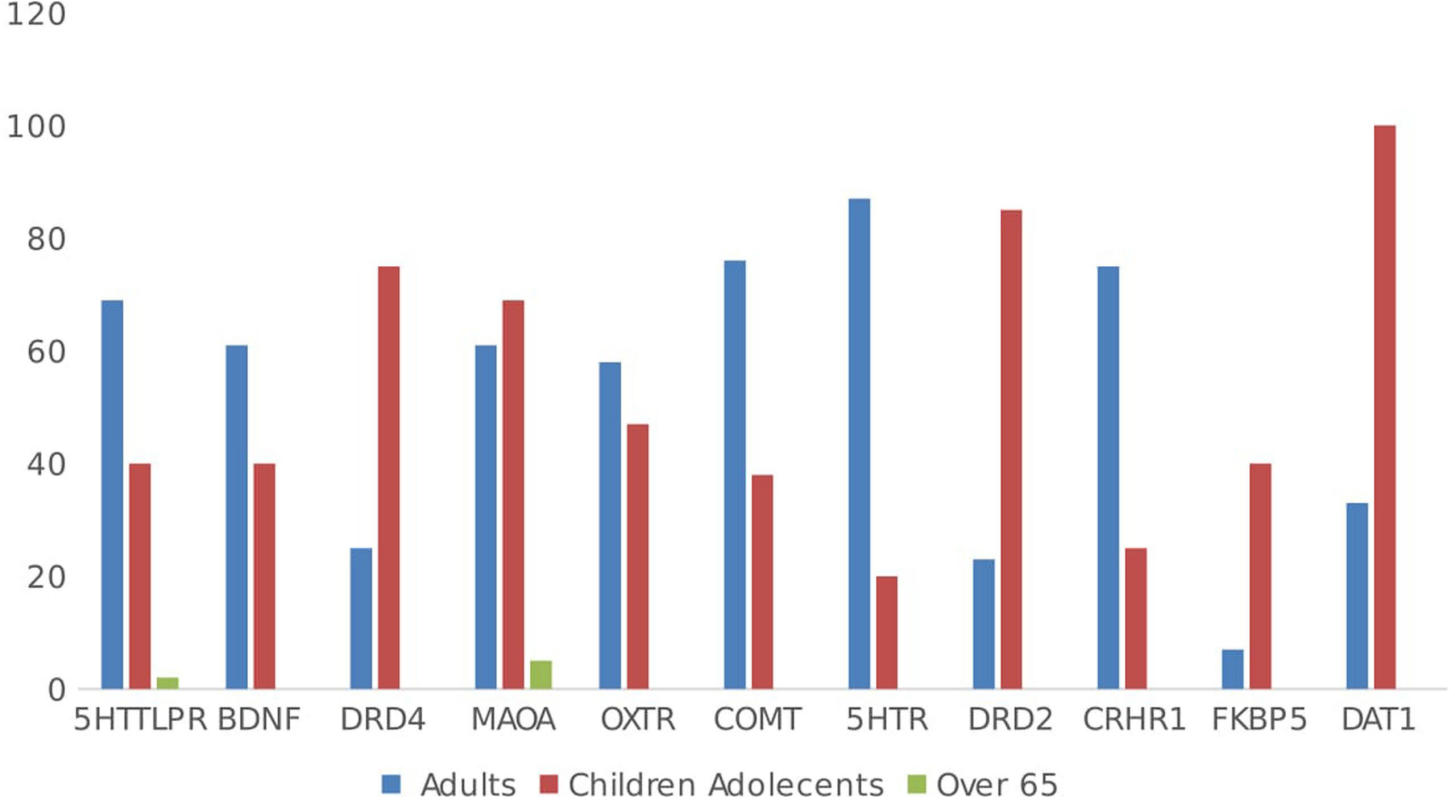
Sample age by gene.

In total, 54.6% (*n* = 172) of the studies included adults and 45.4% (*n* = 143) of the studies included children. When considering the age of the samples and polymorphisms, the focus in children has mostly been on dopaminergic genes (DR, MAOA, FKBP5, DAT1), while in adults, research mostly focuses on serotoninergic-related polymorphisms (5HTT, BDNF, COMT, 5HTR, CRHR1) (see Figure 7). For the individual genes, DRD4 research has predominantly focused on children and adolescents 27 papers include children and adolescents in their samples (75%), while nine papers had adult-only samples (25%). Regarding the 5HTTLPR polymorphism, 61.5% (*n* = 112/182) of articles included adult samples, while 35.7% (*n* = 65/182) included children and adolescents. In total, 81.1% (*n* = 116/143) of GxE papers using child and adolescent samples found positive results; in young adults, the proportion was 77.2% (*n* = 159/206), while 62.5% (*n* = 5/8) of GxE studies using samples of adults or older adults reported positive findings, χ^2^(2, *n* = 357) = 2.00, ns.

### Methodological Considerations

**Figure 8:**
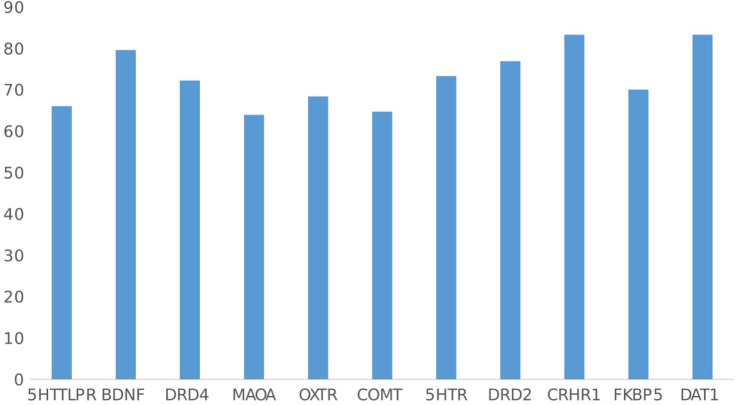
Percentage of gene–environment interactions with positive findings by gene.

Most of the articles showed positive results for GxE, with positive findings ranging from 63.8% for MAOA studies to 83.3% of studies including DAT1 and CRHR1 (mean 72.9% of all articles included) (see Figure 8). Although the consistency of positive findings may be interpreted as congruent with the social susceptibility hypothesis, this could also be a result of publication bias (47). This implies that certain genes make people more susceptible to environment in general, not only to negative environment, as the vulnerability to stress model, but also to environment in general.

**Figure 9:**
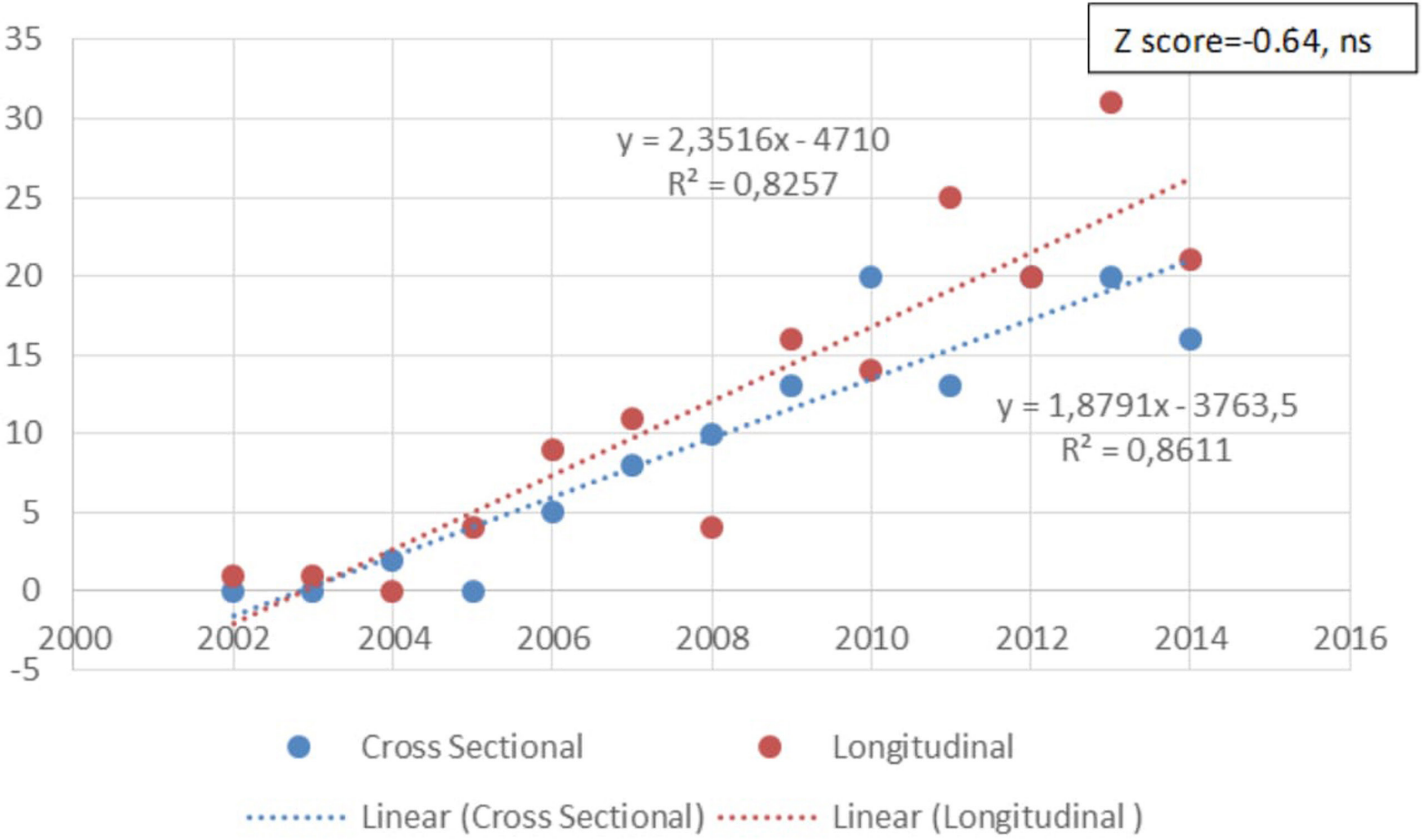
Change in the number of studies of different type (cross-sectional or longitudinal) over time. There is a tendency to increase longitudinal over cross-sectional studies designs over time, but it is not significant (*z* score = 0.64, ns).

The quality of the studies is an important consideration in trying to eliminate false positives in GxE studies. One indication of the quality of studies is the nature of the design. Only 11.4% (*n* = 36/315) of the articles included in this review were experimental in nature; 39.4% (*n* = 124/315) were cross-sectional studies, rendering interpretation of causality difficult. Somewhat more encouraging is that 48.9% (*n* = 154/315) of the identified papers were longitudinal in nature. Furthermore, it is also encouraging that there are a growing number of longitudinal prospective studies and a decreasing focus on cross-sectional studies (see Figure 9), although this latter trend was not significant (*z* score = −0.64, ns).

**Figure 10:**
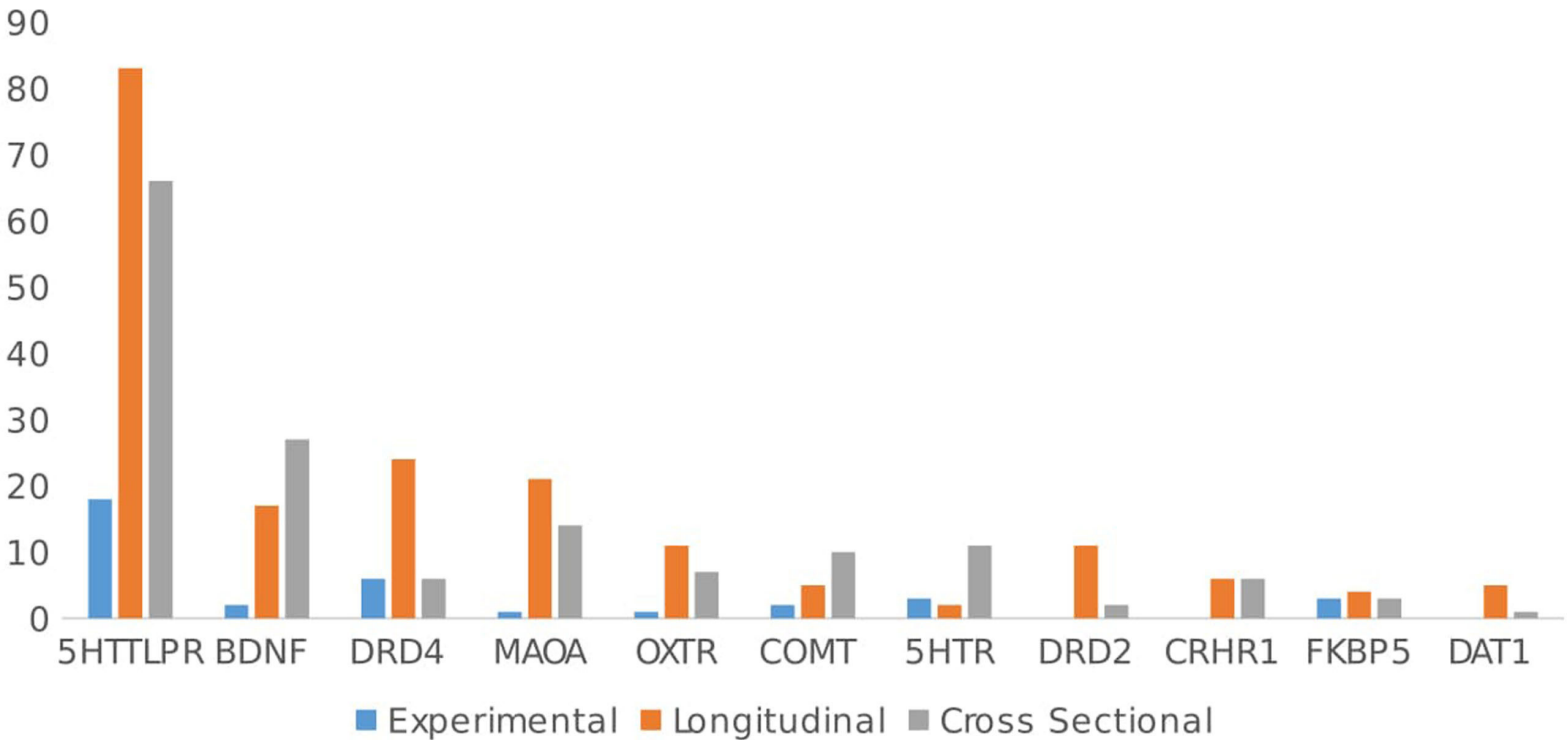
Articles type by gene.

Importantly, for the genes studied most often, that is, those related to serotoninergic function (e.g., 5HTT, BDNF, 5HTR), about half of the articles were cross-sectional in design (45.4%), the rest being experimental (10%) and longitudinal (44.5%). By contrast, dopaminergic and oxytonergic genes (DAT1, MAOA, DR, OXTR, OXT) have been investigated more in longitudinal studies (66%). This difference in the distribution of study design by gene studied is significant χ^2^(2, *N* = 341) = 4.09, *p* < 0.05. 5HTT studies are more cross-sectional, and OXT and DOPA studies are more longitudinal; this is congruent with the assumption that the latter genes are implicated in parenting and may play a crucial role in determining developmental pathways related to attachment and behavioral problems (see Figure 10).

**Figure 11:**
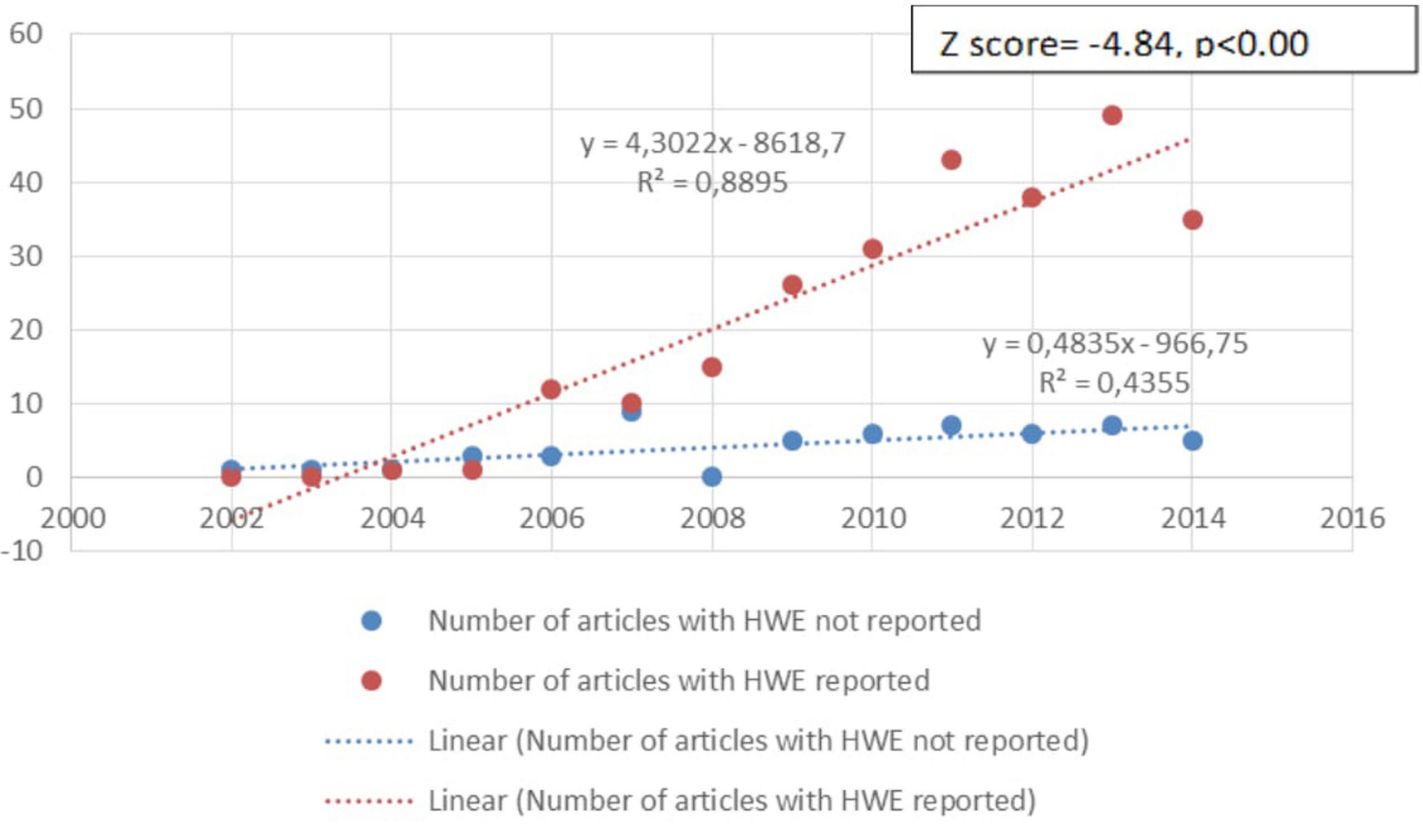
Change over time in the quality of genotyping [reported Hardy–Weinberg equilibrium (HWE)] used in studies. The number of articles that reported HWE was increasing over the years. The first study to report HWE was in 2004. In recent years only 12.5% (2014 *n* = 5/40, 2015 *n* = 7/56) of the studies did not report HWE.

Another criterion that we analyzed was whether studies met the minimum quality criteria in their reporting of the assessment of polymorphisms. Current guidelines (48–52) suggest that the genotyping success rate should be 95% or higher and that the study should report the HWE, linkage equilibrium, or deviations of HWE. Of the 315 articles included in this review, 54 (17.1%) did not report HWE. Most of these studies were earlier studies (see Figure 11). Further, there was no association between studies meeting these quality criteria and positive findings concerning GxE, with 77.3% of studies that reported HWE reporting evidence for GxE and 79.6% of the studies that did not report HWE reporting evidence for GxE, χ^2^(1, *n* = 315) = 0.18, ns.

## Discussion and Recommendations for Future Research

This review demonstrates that research on GxE is clearly flourishing. We have organized the discussion of the findings of this systematic review around six major areas that we believe emerge from the review. For each of these areas, we also discuss guidelines for future research (see Table 2).

**Table 2:** Recommendations for future research.

1. There is a need for standardized genotyping techniques in order to make data from different studies comparable. Minimum quality criteria would include genotyping success rate of 95% or higher and reporting of Hardy–Weinberg Equilibrium (HWE), linkage equilibrium, or deviations of HWE
2. There is a need for standardized assessment of environmental factors, with more attention to gene–environment interactions (GxE) and gene–environment correlations
3. Future research should incorporate “differential susceptibility” or “plasticity” models in order to measure not only the presence/absence of disease or environmental stress but also the “positive” side of human functioning such as the subjective well-being and social support
4. There is a need to move away from candidate genes to general indices of vulnerability/susceptibility genotypes
5. There is a need for a transdiagnostic approach, congruent with the Research Domain Criteria approach, focusing on behavioral systems and pathways involved in GxE
6. There is a need for more studies on the neurobiological mechanisms involved, particularly in humans
7. There is a need to broaden the scope in terms of samples and environments (including culture and developmental context). This will necessarily lead to a greater need and emphasis on longitudinal studies
8. Given the evidence that genes seem to be involved in regulating the effects of environmental influences, further studies are needed investigating the role of genes in explaining response to psychosocial interventions
9. Since there is some evidence for gene–culture interaction in the prediction of social behavior, future studies should incorporate variables that measure cultural aspects, such as individualism/collectivism or ethnicity

### Minimum Quality Assessment of Genes and Environment

First, this review clearly shows that the past decades have witnessed a marked increase in the number of GxE studies. Importantly, it is also evident that the quality of studies in this area is clearly growing, as demonstrated by the relative increase in the numbers of experimental and prospective studies, as well as the growing quality of genotyping in more recent studies. This trend needs to continue, as only well-conducted prospective and experimental studies have the potential to truly increase our knowledge of the role of GxE in explaining vulnerability for psychopathology and the mechanisms involved (see Table 2, point 1). A specific difficulty for the retrospective assessment of environmental factors in GxE studies is that participants’ memories of events may be influenced by genes and that these same genes may influence their personality and behavior. This implies that some retrospective environmental measures may be confused with disorder-relevant genes and so cannot pass the test of rGE (11) (see Table 2, point 2). As noted, one of the major knowledge gaps in the study of mental disorders concerns how an environmental factor external to the person “gets under the skin” to result in a given behavior or mental disorder. Experimental studies of the effect of GxE on neurobiological systems promise to allow us to better understand how environmental and biological factors interact to shape human behavior. Unfortunately, there are very few experimental studies of GxE, with most of these studies finding evidence for GxE effects (85.2%, *n* = 23/27).

### Differential Susceptibility vs. Diathesis Stress

Second, in line with a number of meta-analyses in human and animal research, the majority of GxE studies reported positive results, which were found in around 60–80% of studies (depending on the gene studied); this was the case regardless of whether positive or negative outcomes were focused upon. These findings provide support not just for the role of GxE in human behavior but specifically for social susceptibility rather than vulnerability theories (see Table 2, point 3). As explained earlier, social susceptibility models contend that there are differences between individuals in susceptibility to environmental influences, with some individuals being far more affected than others by both negative and positive contextual conditions. Therefore, one would expect that GxE evidence would be found for both positive and negative circumstances. In contrast, in the vulnerability model, one would expect evidence for GxE only in interaction with negative circumstances. Even though the first study of GxE under the social susceptibility model dates back to 2006 (53), by 2010, only four studies had been published (26, 28, 29, 54). Although we cannot ignore that there is a publication bias, most of the articles published in scientific journals are those with positive results; hence, if we are interested on finding the percentage of positive results, we will find around 70% of positive results for all investigations (47), but if the case was that we cannot trust what is published, we cannot be sure of the evidence for scientific statements, as the usefulness of psychotherapy or psychotropic drugs to treat mental disorders. Furthermore, it is common sense to think that if negative events affect people differently, positive events also will. Therefore, more research is needed in this area, and future studies should include both positive and negative environments and outcomes, rather than a focus on one type of environment or outcome alone, as is typical of most current studies in this area.

### Candidate Gene vs. General Indices of Vulnerability/Susceptibility Genotypes

Third, over half of the studies on GxE have focused on 5HTTPLR. It is clear that there are good reasons for this, as there is good evidence from both animal and human studies that this gene is implicated in susceptibility for environmental influences (3, 55–60). However, as pointed out by others (25), the frequency of the genes studied may not necessarily mean that these genes are the most promising but might partly be a consequence of early positive studies, as for 5HTTPLR. Similarly, although there is a clear scientific rationale for the role of genes related to oxytocin in parenting, as studies have suggested that the oxytocin system plays an important role in social affiliation, the “popularity” of some genes in certain areas (e.g., stress sensitivity vs. parenting) may be partly attributed to the fact that early studies of these genes reported positive findings. It may now be time to take stock of the field and reconsider some of these foci. For instance, although most studies of the 5HTT gene have focused on its influence on depression, increased stress reactivity probably characterizes many types of psychopathology, such as borderline personality disorder or post-traumatic stress disorder, for instance (61–63). In addition, extensive research in both humans and animals has demonstrated structural and functional relationships between the stress system and the reward/affiliation system (64). Furthermore, a known association between a particular genetic polymorphism and a disorder can nominate a gene for a GxE hypothesis, but the absence of such an association does not in itself disqualify a gene (11). In this context, recent work concerning the mapping of the human genome presents another exciting development that needs to be incorporated into future research on GxE. Hence, an exclusive focus on the influence of specific genes in specific disorders or behaviors may be misguided. Instead, genotyping of an array of genes as an index of social susceptibility or a polygenic risk score is likely to be more appropriate when studying complex human behaviors (25) (see Table 2, point 4). Hence, in line with the Research Domain Criteria matrix of the US National Institute of Mental Health (see Table 2, point 5), it may be time to adopt a spectrum approach that cuts across disorders and behaviors, rather than to focus on specific disorders, specific behaviors, or specific outcomes (65, 66). It appears that biological findings for mental disorders are relatively non-specific; most genetic findings and neural circuitry maps appear to link to many different syndromes (67). Until recently, relatively few studies have addressed the question of whether several disorders may share important etiological factors. A transdiagnostic view, considering a more etiologically based approach, is in line with an increasingly comprehensive body of research in genetics, neuroscience, and behavioral and evolutionary science that has transformed the understanding of how the brain produces adaptive behavior and the ways in which normal brain functioning may become disrupted (68). As noted by many (25, 69), such studies will necessitate large samples.

The fact that other trends besides scientific arguments are driving some of the research on GxE is also exemplified by the finding that approximately four times as many articles have focused on 5HTT as on the second most studied gene, BDNF. Serotoninergic alleles (5HTT), predominantly, have been studied with regard to their interaction with early and negative events to predict depression, in longitudinal or cross-sectional studies in adults. Only recently, studies concerning this polymorphism (5HTTLPR) have begun to focus on its interaction with positive events and its underlying neurobiology.

In contrast, dopaminergic alleles have been investigated in studies that address how the genes’ interaction with early positive and negative events predict changes in social behavior in longitudinal or experimental studies in children or adolescents. Yet, these genes may also be important in terms of their interaction with life events in the prediction of psychopathology, particularly as these genes may play a key role in the regulation of the reward system, which has been implicated in depression and substance abuse disorders, for instance (70–73). This further suggests that it may be time for research to move away from candidate genes toward general indices of vulnerability/susceptibility genotypes (25).

### Neurobiological Mechanisms Involved in GxE Should Be Included in Studies

Fourth, most research to date has focused on psychopathology and social behavior. It may now be time to shift more toward the study of the mechanisms involved in GxE. Future studies should routinely include a focus on mechanisms, rather than focusing on GxE alone (see Table 2, point 6). For example, studying if the carriers of plastic alleles are more sensible to experience by having a more reactive hypothalamic-pituitary-adrenocortical axis, which is more susceptible due to epigenetic modification on specific brain areas (74, 75).

### Need for Lifetime Perspective

Fifth, more studies in children and adolescents are needed (see Table 2, point 7). Developmental neuroscience has shown that there are periods of increased plasticity of the brain throughout development. During such periods, experiences may have profound programming and organizing effects on the brain (76, 77). These critical periods refer to time windows where expected experiences are necessary for a particular brain function to develop normally. However, during such times of heightened plasticity, the brain may also be particularly sensitive to negative or positive experiences (78). These critical windows are directly relevant to early prevention and intervention strategies. It may be the case that GxE has a greater impact on children and young adults, while in older adults, the influence of the environment is less dependent on genetic variance.

### Cultural and Ethnic Variables Should Be Included in GxE Studies

Sixth, most of the GxE studies covered in this review have focused on early environment and negative environments in particular. This focus is clearly warranted in view of findings concerning the “programming” of stress and other neurobiological systems by early adversity (78, 79). Yet, as the number of studies finding evidence for GxE in interaction with positive environments equals to negative environmental factors, future studies might do well to simultaneously focus on interactions with both positive and negative environments (see Table 2 point 8). Indeed, if a potentially disadvantageous gene variant is maintained at a high prevalence, this might imply that natural selection has not been able to eliminate the variant because its effects on the phenotype are expressed only under certain environmental conditions and/or perhaps even because it confers an advantage under particular environmental conditions (11). The importance of including recent and positive events in GxE studies is that transforming the environment into a positive one, whether at personal level (i.e., by encouraging prosocial behaviors and psychotherapy interventions), or at political level (i.e., by lobbying for a wider, more positive environment for populations), could have positive outcomes, especially for more sensitive individuals (80).

Further, most studies to date focus on discrete events. However, there is good evidence to suggest that more chronic stressors and broader environmental factors, such as cultural minority status, social disadvantage, and sociocultural factors more generally, may influence GxE (see Table 2, point 9). This may be particularly relevant as there is a clear cultural bias in GxE studies, with almost 90% of studies to date focusing on North American and European populations. Given the potential of gene–culture interactions and even gene–culture co-evolution (81), it is surprising that only a small minority of studies has been conducted in other geographical regions such as Latin America, Africa, and Asia, particularly, as many cultures within these regions are traditionally seen as more interdependent—and thus individuals within these cultures may be more susceptible to environmental factors such as social support. Therefore, cross-cultural studies are needed. This is all the more needed as the prevalence of social susceptibility polymorphisms may vary greatly among different populations; this may reflect a process of natural selection of gene–culture co-evolution, such that genes that serve survival and adaptation in a given culture are selected for. Researchers in the field of cultural neuroscience have argued that maybe the different beliefs, values, and practices of different cultures may influence the selection of genes and interact with genetic variables to regulate human brain and behavior (81, 82). These models suggest that cultural influences may dramatically affect the rate of change of allele frequencies in response to selection (81). For instance, social susceptibility genes (5HTT, OPRM1, MAOA) have been shown to be more prevalent in collectivistic cultures (83), and collectivistic values have been found to moderate the prevalence of depression, for instance, in these cultures (84). Hence, the same polymorphism may interact in different ways in different populations, and therefore, it may not be possible to generalize across different populations. Because of this, caution is needed when attempting to interpret findings on GxE; this is even more the case because these studies are also limited in terms of the types of environmental factors they have studied. Finally, the strong overlap in studies, with only 160 original samples included in this study, and most samples/studies originating in the US and Western Europe are a reason for concern and emphasize the need for caution in drawing conclusions concerning GxE effects.

## Author Contributions

CL and PL were responsible for the design of this study, interpretation of results, and writing. CL and AB were responsible for data extraction. JS prepared the figures and PL and JPJ made final corrections. PL, JS, and JPJ obtained funding (described below). All authors reviewed and accepted the final version.

## Conflict of Interest Statement

The authors declare that the research was conducted in the absence of any commercial or financial relationships that could be construed as a potential conflict of interest.
