# Supplementary material for: Vulnerability or Sensitivity to the Environment? Methodological Issues, Trends, and Recommendations in Gene–Environment Interactions Research in Human Behavior
Source: Front Psychiatry. 2017 Jun 15;8:106. doi: 10.3389/fpsyt.2017.00106 (PMC5475387; doi:10.3389/fpsyt.2017.00106)
Supplement: Supplementary file 1 [file Table_1.docx]

Supplementary Material

# Vulnerability or Sensitivity to the environment?

# Methodological issues, trends, and recommendations in GxE research in human behaviour

Caroline Leighton*, Alberto Botto, Jaime Silva, Juan Pablo Jimenez, Patrick Luyten

*** Correspondence:** Corresponding Author: [carolineleightongeddes@yahoo.com.ar](mailto:carolineleightongeddes@yahoo.com.ar)

# Table 3 Articles included on the review (315)

| Reference | Gene/Variants | Phenotype | Environmental | Country sample |
| --- | --- | --- | --- | --- |
| Caspi A, 2002 | MAOA-uVNTR | Antisocial behavior | Child maltreatment | NZ |
| Caspi A, 2003 | 5HTTLPR | Depression + suicidality | Life stress + child maltreatment | NZ |
| Eley TC, 2004 | 5HTTPLR/5HTR2A T102C /5HTR2C allele 1 /MAOA-uVNTR /TPH | Depression | Environmental risk group | UK |
| Kaufman J, 2004 | 5HTTLPR | Depression | Social supports + maltreatment | USA |
| Fox NA, 2005 | 5HTTLPR | Inhibited behavior | Social support | USA |
| Gillespie NA, 2005 | 5HTTLPR | Depression | Stressful life events | Australia |
| Kendler KS, 2005 | 5HTTLPR | Depression + generalized anxiety | Stressful recent life events | USA |
| Haberstick BC, 2005 | MAOA-uVNTR | Conduct problems + criminal convictions | Child maltreatment | USA |
| Nilsson KW, 2006 | MAOA-uVNTR | Criminal activity | Psychosocial factors | Sweden |
| Mandelli L, 2006 | 5HTTLPR/COMT/5HT1AT102C / DRD4 7R+ | Onset of mood disorders | Stressful recent life events | Italy |
| Surtees PG, 2006 | 5HTTLPR | Depressive | Social adversity | UK |
| Covault J, 2006 | 5HTTLPR | Drinking and drug use | Stressful recent life events | USA |
| Kim-Cohen J, 2006 | MAOA-uVNTR | Development of psychopathology | Exposure to physical abuse | UK |
| Taylor SE, 2006 | 5HTTLPR | Depressive symptomatology | Stressful early family environment and recent | USA |
| Jacobs N, 2006 | 5HTTLPRrs25531 | Depression | Negative affectivity (neuroticism)/stressful recent life events | Belgium |
| Zalsman G, 2006 | 5HTTLPRrs25531 | Depression and suicidality | Stressful recent life events | USA |
| Kaufman J, 2006 | 5HTTLPR/BDNF Val66Met | Depression | Child maltreatment + social supports | USA |
| Sjöberg RL, 2006 | 5HTTLPR | Depression | Combination of psychosocial variables | Sweden |
| Bakermans-Kranenburg MJ, 2006 | DRD4 7R+ | Externalizing behavior in preschoolers | Observed maternal sensitivity | Netherlands |
| Wilhelm K, 2006 | 5HTTLPR | Depression | Negative and positive recent life events | Australia |
| Jacobs RH, 2006 | 5HTTLPR | Depression | Recent Life events/Neuroticism | USA |
| Widom CS, 2006 | MAOA-uVNTR | Violent and antisocial behavior | Child maltreatment | USA |
| Brocke B, 2006 | 5HTTLPR | Startle magnitudes response to intense noise | Pictures of unpleasant, pleasant and neutral valance | Germany |
| Frazzetto G, 2007 | MAOA-uVNTR | Physical aggression | Early Traumatic Life events | Italy |
| Cicchetti D, 2007 | 5HTTLPR/MAOA-uVNTR | Depressive symptomatology | Child maltreatment | USA |
| Gervai J, 2007 | DRD4 7R+ | Infant attachment | Affective-disrupted communication between mother & infant | Hungry/USA |
| Dick DM, 2007 | 5HTTLPR | depression + alcohol dependence | Current stress | USA |
| Oreland L, 2007 | MAOA-uVNTR | Criminal activity | Good/bad psychosocial environment | Sweden |
| Kilpatrick DG, 2007 | 5HTTLPR rs25531 | PTSD and depression | Hurricane exposure and low social support | USA |
| Cervilla JA, 2007 | 5HTTLPR | Depression | Stressful recent life events | Spain |
| Scheid JM, 2007 | 5HTTLPR | Depressive symptoms | Recent and early Stressful events | USA |
| Steiger H, 2007 | 5HTTLPR rs25531 | Dimensional personality disturbances + borderline personality disorder | History of sexual or physical abuse | Canada |
| Chipman P, 2007 | 5HTTLPR | Depression | Child maltreatment + stressful recent life events | Australia |
| Chorbov VM, 2007 | 5HTTLPR rs25531 | Depression | Recent and early stressful events | USA |
| Beaver KM, 2007 | DRD2 TaqIA | Adolescent victimization | Recent stressful events | USA |
| Mills-Koonce WR, 2007 | DRD2 TaqIA | Child affective problems | Maternal sensitivity observed | USA |
| Nilsson KW, 2007 | MAOA-uVNTR | Behaviour during alcohol consumption | Child maltreatment | Sweden |
| Sjöberg RL, 2007 | MAOA-uVNTR | Criminal activity | Psychosocial factors | Sweden |
| Keltikangas-Järvinen L, 2007 | TPH1 A779C / A218C | Harm avoidance | Child environment | Finland |
| Kaufman J, 2007 | 5HTTLPR | Alcohol use | Child maltreatment, family loading OH + substance use | USA |
| Kim JM, 2007 | 5HTTLPR/BDNF | Depression | Stressful recent life events | Korea |
| Sheese BE, 2007 | DRD4 7R+ | Temperament in early childhood | Parenting observed | USA |
| Bakermans-Kranenburg MJ, 2008 | 5HTTLPR/OXTR rs53576 | Observed maternal sensitivity | Maternal depression + marital discord | Netherlands |
| Brummett BH, 2008 | 5HTTLPR | Depression | Care giving status + low childhood socioeconomic status (SES) | USA |
| Guo G, 2008 | DAT1 9R/10R | Number of sexual partners 18-26 y | Psychosocial combined recent context | USA |
| Wichers M, 2008 | BDNF val66met | Negative or positive effect in response to daily events | Event stress and social stress in daily life | Belgium |
| Ducci F, 2008 | MAOA-uVNTR + 9 SNPs MAOA/MAOB | Alcoholism + antisocial personality disorder | Child sexual abuse | USA native tribe |
| Gilissen R, 2008 | 5HTTLPR | Electrodermal reactivity | Children's attachment representation | Netherlands |
| Wichers M, 2008 | 5HTTLPR/BDNF val66met | Depressive symptoms | Child adversity | Belgium |
| Bakermans-Kranenburg MJ, 2008 | DRD4 7R+ | Externalizing behavior | Intervention sessions focusing on maternal sensitivity and discipline | Netherlands |
| Lazary J, 2008 | 5HTTLPR + SNP: rs2020942, rs140700, rs3798908, rs1042173 | Depression | Threatening life events | Hungry |
| Wray NR, 2008 | 5HTTLPR rs25531 | Depression | Stressful recent life events | Australia |
| Nilsson KW, 2008 | MAOA-uVNTR | OH problem behavior | Family relations + child maltreatment | Sweden |
| Bradley RG, 2008 | CRHR1 rs110402, rs7209436 | Depression | Child maltreatment | USA |
| Binder EB, 2008 | FKBP5 rs4713916, rs1360780, rs3800373 + 5 SNPs  CRHR1 rs110402, rs242924, rs7209436 | PTSD | Child maltreatment | USA |
| Steiger H, 2008 | 5HTTLPR rs25531 | Emotional dysregulation, dissocial behavior, inhibition,  and compulsivity | Child maltreatment | Canada |
| Bakermans-Kranenburg MJ, 2008 | DRD4 7R+ | Daily cortisol | Attachment based intervention | Netherlands |
| Alexander N, 2009 | 5HTTLPR rs25531 | HPA axis responses | Stressful recent life events | Germany |
| Armbruster D, 2009 | 5HTTLPR rs25531 | Startle magnitudes in response to intense noise | Stressful recent life events + pictures of unpleasant, pleasant and neutral valence | Germany |
| Araya R, 2009 | 5HTTLPR rs25531 | Emotional symptoms | Stressful life events + Maternal postnatal depression at 2 or 8 months | UK |
| Aslund C, 2009 | 5HTTLPR | Depression | Child maltreatment | Sweden |
| Brody GH, 2009 | 5HTTLPR | Risk behaviors across preadolescence | Preventive intervention | USA |
| Brody GH, 2009 | 5HTTLPR | Substance use | Parenting Involved | USA |
| Brody GH, 2009 | 5HTTLPR | Risk behavior | Preventive intervention | USA |
| DiLalla, 2009 | DRD4 7R+ | Aggressive and prosocial behaviors | Environmental influences from parents + peers aggression | USA |
| Aguilera M, 2009 | 5HTTLPR/BDNF BDNF val66met | Depressive symptoms | Early adversity | Spain |
| Stevens SE, 2009 | DRD4 7R+ /DAT1 haplotype 40-bp VNTR 3'UTR & 30-bp VNTR intron 8 | ADHD | Severe early institutional deprivation | UK |
| Beaver KM, 2009 | MAOA-uVNTR | Fraudulent behaviors | Exposure to delinquent peers | USA |
| Amstadter AB, 2009 | RGS2 rs4606 | Suicidal ideation | Social Support + Hurricane 2004 | USA |
| Dick DM, 2009 | GABRA2 rs497068 rs548583 rs279871 rs279858 rs279845 rs1440130 rs279826 rs279827 rs279828 rs279836 | Trajectories of externalizing behavior | Parental monitoring | USA |
| Laucht M, 2009 | 5HTTLPR rs25531 | Alcohol consumption | Family adversity/negative recent life events | Germany |
| Laucht M, 2009 | 5HTTLPR rs25531 | Depression and anxiety | Family adversity/negative recent life events | Germany |
| Bukh JD, 2009 | 5HTTLPR rs25531 /BDNFVal66Met /COMTVal158Met /ACE 1 rs4291 /TPH1A218C /5HTR1A C-1019G /5HTR2A T102C /5HTR2Crs3813929 | Depression | Stressful recent life events | Denmark |
| Polanczyk G, 2009 | CRHR1TAT haplotype rs7209436, rs110402, rs242924 | Depression | Child maltreatment | UK/NZ |
| Spangler G, 2009 | 5HTTLPR/ DRD4 7R+ ,*-*521 C/T SNP | Attachment | Early maternal caregiving | Germany |
| Bet PM, 2009 | GR NR3C1 22/23EK- 9beta- BclI | Depression + morning cortisol | Child adversity | Netherlands |
| Ritchie K, 2009 | 5HTTLPR rs25531 | Depression | Adverse and protective childhood events | France |
| Kochanska G, 2009 | 5HTTLPR | Self-regulation | Early mother-child relationship | USA |
| Amstadter AB, 2009 | RGS2 rs4606 | PTSD | Social support | USA |
| Weder N, 2009 | MAOA-uVNTR | Aggressive behavior | Child maltreatment | USA |
| Kinnally EL, 2009 | MAOA-uVNTR | Trait impulsivity + aggression depression | Parental care/child stressors | USA |
| Koenen KC, 2009 | 5HTTLPR rs25531 | PTSD | County level social + environment + hurricane | USA |
| Tyrka AR, 2009 | CRHR1 rs110402 rs242924 | HPA reactivity to DEX | Child maltreatment | USA |
| Gatt JM, 2009 | BDNF Val66Met. | Amygdala–hippocampal– prefrontal grey matter volume + heart rate + temperament | Early life stress | BRAIN RESOURCE INTERNATIONAL DATABASE |
| Zhang K, 2009 | 5HTTLPR, STin2 5-HTT/ 5HTR1A rs6295/ HTR1B rs130058, rs11568817/ HTR2A T102C (rs6313) | Depression | Negative life events + social supports | China |
| Veletza S, 2009 | 5HTTLPR | Depression | Stressful recent + early life events | Greece |
| Xie P, 2009 | 5HTTLPR rs25531 | PTSD + substance dependence | Child adversity, adult traumatic events | USA |
| Wagner S, 2009 | 5HTTLPR | Impulsive aggression BPD | Traumatic life events | Germany |
| Larsen H, 2010 | DRD4 7R+ | Alcohol consumption | Alcohol related cues | Netherlands |
| Edwards AC, 2010 | MAOA-uVNTR | Externalizing behavior | Early physical discipline before 6 y | USA |
| Conway CC, 2010 | 5HTTLPR rs25531 /COMT Val158Met | Depression | Chronic family Stress | Australia |
| Coventry WL, 2010 | 5HTTLPRrs25531 | Depression + suicidality | Stressful recent life events | Australia |
| Kumsta R, 2010 | 5HTTLPR rs25531 | Emotional problems in adolescence | Early institution deprivation + life events | Rumania/UK |
| Li JJ, 2010 | 5HTTLPR | Antisocial behavior | Child maltreatment | USA |
| Nederhof E, 2010 | 5HTTLPR rs25531/ BDNFval66met rs6265, rs25531 | Effortful control | Child adversity | Netherlands |
| Nederhof E, 2010 | 5HTTLPR rs25531/ BDNFval66met rs6265, rs25531 | Depression | Child adversity | Netherlands |
| Van Der Zwaluw CS, 2010 | DRD2 Taq1A (rs1800497) | Alcohol use | Parental rules | Netherlands |
| Sugden K, 2010 | 5HTTLPR | Emotional problems | Bullying victimization | UK |
| Grabe HJ, 2010 | CRHR1 rs17689882 | Depression | Child maltreatment | Germany |
| Power T, 2010 | 5HTTLPRrs25531 | Depression | Stressful recent life events | France |
| Uddin M, 2010 | 5HTTLPR | Depressive symptoms | Poor social environments | USA |
| Benjet C, 2010 | 5HTTLPR | Depressive symptoms | Victimization bullying | USA |
| Wagner S, 2010 | COMT Val158Met | Impulsive aggression | Traumatic life events | Germany |
| Wagner S, 2010 | BDNF val66met | Impulsive aggression | Traumatic life events | Germany |
| Goldman N, 2010 | 5HTTLPR | Depressive symptoms | Early + recent stressful life events | Taiwan |
| Pluess M, 2010 | 5HTTLPR | Neuroticism | Recent stressful life events | USA |
| Ressler KJ, 2010 | 5HTTLPRrs25531 /CRHR1 rs7209436, rs4792887 rs110402 | Depressive symptoms | Child abuse | USA |
| Saiz PA, 2010 | 5HTR2AA-1438G (rs6311)/STin2 VNTR 5-HTTLPR | Personality traits | Early life events | Spain |
| Hayden Ep, 2010 | DRD2 TaqIA | Depressive + anxious symptoms | Parental support/ intrusion | Canada |
| Hayden Ep, 2010 | BDNF val66met | Negative emotionality in the child | Parental depression + parental discord | Canada |
| Roy A, 2010, 2010 | FKBP5rs3800373, rs9296158, rs1360780, rs9470080 | Risk for attempting suicide | Child trauma | USA |
| Gatt JM, 2010 | 5HTR3A rs1062613 (C178T) | Emotional brain networks + depression | Early life trauma | BRAIN RESOURCE INTERNATIONAL DATABASE |
| Wagner S, 2010 | COMT Val158Met | Impulsive in female BPD | Traumatic life events | Germany |
| Antypa N, 2010 | 5HTTLPR rs25531 | Cognitive vulnerability +  Cognitive reactivity | Child emotional abuse | Netherlands |
| Frodl T, 2010 | 5HTTLPR rs25531 | Reduced hippocampal volumes + other brain alts | Child trauma | Germany |
| Kim HK, 2010 | 5HTR1A C-1019G | Depression | Recent negative life stressors | Korea |
| Luijk MP, 2010 | BclI (rs4142347), TthIIII (rs10052957), GR-9b (rs6198), N363S (rs6195), ER22/23EK (rs6189/6190)- FKBP5 (rs1360780) | Cortisol reactivity | Attachment (positive and negative valence) | Netherlands |
| Lazary J, 2010 | 5HTTLPR rs2020942, rs140700, rs3794808, rs104217 | Depressive phenotype | Threatening life events | Hungry |
| Middeldorp CM, 2010 | 5HTTLPR | Anxious depression + neuroticism | Number recent and early negative Life Event | Netherlands |
| Blaya C, 2010 | 5HTTLPR rs25531/ 5HTR1A rs6311/ 5HTR2AT102C | Panic disorder | Child trauma + parenting | Brazil |
| Wagner S, 2010 | COMT Val158Met | Impulsive aggression | Traumatic life events | Germany |
| Way BM, 2010 | 5HTTLPR | Cortisol responses | TSST with ≠ audience | USA |
| van Roekel E, 2010 | 5HTTLPR | Loneliness | Parental support | Netherlands |
| Kim HS, 2010 | OXTR rs53576 | Sensitivity to cultural norms  Support seeking | Recent stressful life events | USA/Korea |
| van Strien T, 2010 | DRD2 TaqIA | Emotional eating | Parental control | Netherlands |
| Chen FS, 2011 | OXTR rs53576 | Salivary Cortisol and subjective stress | Social Support during experiment | Germany |
| Knafo A, 2011 | DRD4 7R+ | Self-initiated prosocial behavior | Parenting | Israel |
| Gibb BE, 2011 | 5HTTLPR rs25531 | Inferential style | Maternal criticism | USA |
| Latendresse SJ, 2011 | CHRM2 9 SNPs | Adolescent externalizing trajectories | Peer group antisocial behavior | USA |
| Dong L, 2011 | PER1 rs3027172 | Alcohol drinking | Psychosocial Stress and family function prior to birth | Germany |
| Salo J, 2011 | 5HTR2A T102C | Adulthood social attachment | Child maternal nurturance | Finland |
| Lahey BB, 2011 | DAT1 9R/10R | Child conduct disorder symptoms and ADHD | Early maternal parenting | USA |
| Lee SS, 2011 | MAOA-uVNTR | Antisocial behavior | Deviant peer affiliation | USA |
| Burkhouse KL, 2011 | 5HTTLPR rs25531 | Behavioral inhibition in child | Parenting | USA |
| LuijK MP, 2011 | MR BclI  rs41423247/ TthIIII rs10052957/ GR-9B rs6198 / N363S rs6195 / ER22/23EK  rs6189, rs6190 | Attachment | Parenting | Netherlands |
| Witt SH, 2011 | NPYrs16147 | Endocrine stress responses | Early adversity | Germany |
| Fergusson DM, 2011 | MAOA-uVNTR | Antisocial behaviour | Child maltreatment | NZ |
| Fergusson DM, 2011 | 5HTTLPR | Psychiatric disorders | Child maltreatment + recent adverse life events | NZ |
| Luijk MP, 2011 | 5HTTLPR/DRD4 7R+/DRD2 TaqIA /COMT Val158Met /OXTR rs53576, rs2254298 | Attachment | Parenting | USA/Netherland |
| Mehta D, 2011 | 5HTTLPR | Depressive symptoms | Stressful life events + satisfaction with partner | Germany |
| Zimmermann P, 2011 | FKBP5rs3800373, rs1360780, rs4713916rs9296158, rs9470080 | Depression | Adverse life events | Germany |
| Uher R, 2011 | 5HTTLPR | Depression | Child maltreatment | NZ/UK |
| Bakermans-Kranenburg MJ, 2011 | DRD4 7R+/5HTTLPR | Unresolved Loss + Adult trauma | Parental problems | USA |
| Jenness JL, 2011 | 5HTTLPRrs 25531 | Depressive symptoms | Chronic family stress + recent stressful events | USA |
| Mitchell C, 2011 | 5HTTLPRSTin2 VNTR | Depression | Chronic environmental stressor | USA |
| Pluess M, 2011 | 5HTTLPR | Infant negative emotionality | Prenatal maternal anxiety | Netherlands |
| Way BM, 2011 | 5HTTLPR | Cardiovascular reactivity | TSST with ≠ audience P/N | USA |
| Dick DM | CHRM2 9 SNPs | Externalizing behavior | Parental monitoring P/N | USA |
| Ben-Efraim YJ, 2011 | CRHR1 7 SNPs | Suicide attempts | Stressful life events | Ukraine |
| Reiner I, 2011 | DRD4 7R+ | Personality traits | Adverse life events | Germany |
| Carver CS, 2011 | 5HTTLPR | Impulsivityreacting to emotions | Child adversity | USA |
| Carver CS, 2011 | 5HTTLPR/BDNF val/met | Depression | Child adversity | USA |
| Klauke B, 2011 | 5HTTLPRrs25531 | Anxiety sensitivity | Child maltreatment | Germany |
| Heim C, 2011 | CRHR1rs110402 | Depression and cortisol response to DEX | Child trauma | USA |
| Artero S, 2011 | 5HTTLPR rs25531 | Depression | Highly stressful life event | France |
| Carli V, 2011 | 5HTTLPR | Psychological resilience (scale) and depression (HAM) | Child trauma | Italy |
| Mekli K, 2011 | 5HTR1A rs6295, rs878567 /5HTR1Brs6296, rs130058, s11568817 | Depression and anxiety scores | Recent stressful life events / face emotion processing task | UK |
| Shinozaki G, 2011 | 5HTTLPR | Heart rate | Child abuse | USA |
| Hankin BL, 2011 | 5HTTLPR rs25531 | Positive affect and Behavioral Inhibition/Activation System scales | Parenting | USA/Netherland |
| Meng X, 2011 | SLC6A4 rs12449783, rs3794808, rs2020942, rs11080122, rs6354, rs2020939 /MAOA rs2283724 / BDNF rs12273539, rs10835210, rs2030324 | Depression + anxiety | Psychosocial combine index of stressful parenting | China |
| Jacobs RH, 2011 | 5HTTLPR+ STin2 VNTR | Processing of emotional faces | Maternal depressive history | USA |
| Kohen R | 5HTTLPRSTin2 VNTR | Response to Psychosocial Treatment | Psychosocial Treatment in Post-Stroke Depression | USA |
| Mueller A, 2011 | 5HTTLPR rs25531 | Cortisol stress responses | Stressful life events + TSST | Germany |
| Schoebi D, 2011 | 5HTTLPR rs25531 | Spouses’ sensitivity | Before and after marital discussions | USA |
| Armbruster D, 2011 | 5HTR1A 1019 G/C | Cortisol stress responses | Stressful life events + TSST | Germany |
| Lemogne C, 2011 | 5HTTLPR rs25531 | Amygdala reactivity | Life Stress + task Cognitive Appraisal | France |
| Brummett BH, 2011 | 5HTTLPRrs25531 | Cardiovascular reactivity | Caregiver | USA |
| Bakermans-Kranenburg MJ, 2011 | DRD4 7R+ | Donating behavior | Child attachment | Netherlands |
| van Roekel E, 2011 | 5HTTLPR | Depressive symptoms | Parental support/parental depressive fillings | Netherlands |
| Bradley B, 2011 | OXTRrs53576 | Emotional dysregulation + adult attachment | Child maltreatment | USA |
| Das D, 2011 | DRD4 7R+ | Emotional resilience | Child adversity | Australia |
| Fortuna K | DRD4 7R+ | Maternal sensitivity | Child Risk at Birth | Israel |
| van Roekel E | DRD2 TaqIA | Adolescent loneliness | Parental support | Netherlands |
| Nyman ES, 2011 | 5HTTLPR 5 SNPs/ TPH2 11 SNPs/ COMT12 SNPs/ MAOA 6 SNPs /DRD1 4 SNPs /DRD2 14 SNPs /DRD3 13 SNPs /DRD4 2 SNPs /DRD52 SNPs | Depressiveness | Early environmental risk factors | Finland |
| Reif A, 2011 | NOS1 ex1f-VNTR | Impulsivity | Stressful life events and family environment | Estonian |
| Alexander N, 2012 | 5HTTLPR rs25531 | Amygdale reactivity | Stressful/threatening situations | Germany |
| Klauke B, 2012 | COMT Val158Met | startle response | Caffeine placebo-controlled intervention + child maltreatment | Germany |
| Buchmann AF, 2012 | 5HTTLPR rs25531 /BDNF val66met | Depressive symptoms + BDNF level | Early-life adversity | Germany |
| Cicchetti D, 2012 | 5HTTLPR/CRHR1 rs110402, rs242924, rs7209436 /DRD4 C-521T / OXTR rs53576 | Resilience in adaptive functioning | Child maltreatment | USA |
| Conway CC, 2012 | 5HTTLPRrs25531 | Aggression | Chronic and recent stressors | Australia |
| Li JJ, 2012 | DAT1 9R/10R | ADHD symptoms | Child maltreatment | USA |
| Drury SS, 2012 | 5HTTLPR/BDNF val66met | Attachment | Foster care vs institutional rearing | Rumania |
| Gibbons FX, 2012 | 5HTTLPR / DRD4 7R+ | Life history of strategies + associated cognitions | 3 Different sources of recent stress | USA |
| Jonassaint CR, 2012 | 5HTTLPR | Self-esteem | Adolescent environmental conditions | USA |
| Brody GH, 2012 | DRD4 7R+ | Adult drug use trajectory | Recent stressful life events | USA |
| Smith HJ, 2012 | DRD4 7R+ | Effortful control | Parenting observed | Canada |
| Bevilacqua L, 2012 | FKBP5rs3800373, rs9296158, rs1360780, rs9470080 | Aggressive behavior | Child trauma | Italy |
| Li JJ, 2012 | MAOA-uVNTR | Child conduct + ADHD | Parenting P/ N | USA |
| Perea CS | 5HTTPLR STin2 VNTR /5HT1A C(−1019)G / BDNF val66met | Negative affectivity | Stressful life events | Colombia |
| Alasaari JS, 2012 | 5HTTLPR rs25531 | Serotonin transporter gene (SLC6A4) promoter methylation density | High work stress/ Burn out | Finland |
| Vaske J, 2012 | 5HTTLPR rs25531 | Alcohol/THC use problems + general criminal | Child neglect | USA |
| Nederhof E, 2012 | DRD2 TaqIA/ DRD4 7R+ /COMT val158met | Externalizing behavior | Divorced parents before 11 | Netherlands |
| Petersen IT, 2012 | 5HTTLPR rs25531 | Adolescents’ trajectories of anxious/depressed symptoms | Stressful recent Life Events | USA |
| Walsh ND, 2012 | 5HTTLPR rs25531 | Amygdala reactivity | Child adversity | UK |
| Zavos HMS, 2012 | 5HTTLPR rs25531 | Anxiety sensitivity | Stressful recent life events | UK |
| Everaerd D, 2012 | 5HTTLPR rs25531 | Hippocampal volume | Child adversity | Netherlands |
| Owens M, 2012 | 5HTTLPR rs25531 | Cognitive and Emotional Processing | Child adversities | UK |
| Fisher HL, 2012 | 5HTTLPR | Depression | Child maltreatment | UK |
| Tiemeier H, 2012 | 5HTTLPR rs25531 | Child emotional problems | Intrauterine + postnatal environment + maternal anxiety | Netherlands |
| Fisher HL, 2012 | 5HTTLPR | Depression | Stressful recent life events | UK |
| Velders FP, 2012 | GR/FKBP5 rs6189, rs6190, rs10052957, rs41423247, rs6195, rs6198 (rs1360780) | Emotional and behavioral problems + cortisol | Prenatal maternal psychological symptoms | Netherlands |
| Grabe HJ, 2012 | 5HTTLPR rs25531 / BDNF Val66Met | Depression | Child abuse | Germany |
| Grabe HJ, 2012 | 5HTTLPR rs25531 | Depression | Child Abuse and Adult Traumatic Events | Germany |
| Armbruster D, 2012 | COMT Val158Met /BDNF Val66Mett | Cortisol response | Stressful life events + TSST | Germany |
| Quinn CR, 2012 | 5HTTLPR/BDNF Val66Met | Depression | Early stressful life events | BRAIN RESOURCE INTERNATIONAL DATABASE |
| Xie P, 2012 | 5HTTLPR | PTSD | Child adversity | USA |
| Stoltenberg S, 2012 | TPH2 rs1386483/ 5HTTLPR rs25531/MAOA-uVNTR/  HTR1A C-1019G (rs6295)/ HTR1B G861C (rs6296)/ HTR2A T102C (rs6313) | Impulsivity trait | Child trauma | USA |
| Stoltenberg S | 5HTTLPRrs25531 | Eating Problems + impulsiveness | Child trauma | USA |
| Schellekens AF, 2012 | COMTVal158Met / DRD2 Taq1A | Alcohol dependence | Child adversity | Netherlands |
| Troisi A , 2012 | OPRM1A118G | Attachment + personality trait rejection sensitivity | Early maternal care | Italy |
| White MG, 2012 | FKBP5 rs7748266, rs1360780, rs9296158, rs3800373, rs9470080, rs9394309 | Reactivity of the amygdala | Child trauma | USA |
| Beach SRH, 2012 | DRD4 7R+ | Valence of parent-child interactions | Compound chronic stressors/supports | USA |
| Beach SRH , 2012 | 5HTTLPR | DNA methylation at 5HTTLPR | Preadolescent cumulative SES-related risk | USA |
| Chen J, 2012 | BDNF Val66Met | Depression | Stressful life events | China |
| Chen J, 2012 | BDNF Val66Met | Depression | Stressful life events | China |
| Hasler R, 2012 | CREB1rs2709376, rs7569963, rs7594560 rs4675690 | Anger traits + suicidal behaviours | Child sexual abuse | France/Switzerland |
| Kuepper Y, 2012 | 5HTTLPR rs25531 | Neuroticism + life satisfaction | Life positive and negative events | Germany |
| Verhoeven FEA, 2012 | MAOA-uVNTR | Aggression | Child trauma | Netherlands |
| Nikulina V, 2012 | MAOA-uVNTR | Mental health outcomes | Child Abuse and Neglect | USA |
| Belsky , 2013J | 5HTTLPR/DRD4 7R+ | Externalizing problems + social skills | Child-care quality | USA |
| Cutuli JJ, 2013 | 5HTTLPR rs25531 | Depression | Child maltreatment | USA |
| Laucht M, 2013 | CRHR1rs7209436, rs110402, rs242924, rs17689882 | Depressive symptoms | Child adversity | Germany |
| Fandiño-Losada A, 2013 | 5HTTLPR rs25531 | Depression | Early vs recent separation of parents or partners | Sweden |
| Mileva-Seitz V, 2013 | OXT rs2740210 rs4813627 / OXTR rs237885 | Quality of mothering and postpartum mood | Quality of care mothers + child trauma | Canada |
| Brody GH, 2013 | DRD4 7R+ /DRD2rs6279, rs6277, rs6275 ANKK1 /GABRG1rs497565, rs1497571 /GABRA2rs567926,rs534459, rs529826, rs279858 | Alcohol use | Preventive intervention | USA |
| Lucas- Thompson R, 2013 | OXTRrs53576 | PTSS + impaired functioning post Collective stress 11/09/01 | Negative social environment and economic stress | USA |
| Priess-Groben HA, 2013 | 5HTTLPR rs25531/MAOA-uVNTR | Depressive symptoms | Negative life events | USA |
| Haase CM, 2013 | 5HTTLPR | Changes in Marital Satisfaction Over Time | Negative and positive emotional behavior during marital conflict | USA |
| Belsky J, 2013 | DRD4 7R+ | Children’s social functioning | Childcare quality | USA |
| Li JJ, 2013 | 5HTTLPR | Depression + suicide risk | Family support | USA |
| Ming QS, 2013 | 5HTTLPR | Depressive symptoms | Recent negative life events | China |
| Liu B, 2013 | 5HTTLPR | Anxiety | Job-related risk factors | Sweden |
| Nobile M, 2013 | 5HTTLPR rs25531 | Internalizing symptoms | Socioeconomic status + family structure | Italy |
| Li JJ, 2013 | DAT1 9R/10R | ADHD | Parenting behaviors | USA |
| Brown GW, 2013 | 5HTTLPR | Depression | Child maltreatment | UK |
| Sweitzer MM, 2013 | DRD4 7R+ | behavioral endophenotype of Impulsive decision-making | Child socioeconomic status | USA |
| Shinozaki G, 2013 | 5HTR2A A rs7997012 | Suicide attempts | Child abuse | USA |
| Power RA, 2013 | 5HTTLPR | Depression | Child maltreatment, adult stressful life events | UK |
| Peyrot WJ, 2013 | 5HTTLPR rs25531 | Depression | Life time + recent stressful life events | Netherlands |
| Miller S, 2013 | BDNF val66met | Clinical course of Bipolar disorder | Early life stressful events | USA |
| Gallardo-Pujol D, 2013 | MAOA-uVNTR | Aggressive behavior | Experimental recent stressful event (Cyber ball software) | Spain |
| Markus CR, 2013 | 5HTTLPR | Depressive symptoms | Early and recent stressful life events + trait neuroticism | Netherlands |
| Kieling C, 2013 | MAOA-uVNTR | Conduct problems | child maltreatment | Brazil |
| Tomoda A, 2013 | 5HTTLPR | Depression symptoms | Mother’s history of recurrent major depression | Japan |
| Blomeyer D, 2013 | PER2rs56013859 | Alcohol Drinking | Stressful recent Life Events | Germany |
| Berry D, 2013 | DRD4 7R+ | Children’s inattention problems | Early care giving experiences | USA |
| Conley C, 2013 | 5HTTLPR + DRD2 A1 allele taq1A +MAOA-uVNTR | Academic and Behavioral Outcomes | Birth weight discordance within twin pairs | USA |
| Apter-Levy Y, 2013 | OXTR rs2254298 | Child’s Mental Health, Social Engagement, + Empathy | Early maternal depression | Israel |
| Schroeder KB, 2013 | 5HTTLPR/5HTR2A rs2254298 | Cooperative behavior | +/- punishment in experiment economic game task | UK |
| Sokolowski M, 2013 | 113 SNPs located in 24 glutamatergic and GABA genes | Suicide attempts | Child /adolescent physical assault | Ukraine |
| Sasaki JY, 2013 | DRD4 7R+ | Prosocial behavior | Experiment situational prime of religion task | USA |
| Min JA, 2013 | BDNF Val66Met | Anxiety | Child maltreatment | Korea |
| Miranda Jr, 2013 | OPRM 1A118G | Alcohol use disorder | Parenting practices + affiliation with deviant peers | USA |
| McQuaid RJ, 2013 | OXTRrs53576 | Depressive symptoms | Early-life maltreatment | Canada |
| Ramsey H, 2013 | COMT-Val158Met / BDNF-Val66Met | Psychotic experiences | Child trauma | Ireland |
| Perry BL, 2013 | GABRA2 rs279871 | Alcohol dependence | Negative and positive daily life events | USA |
| Baumann C, 2013 | COMT rs468 /MAOA-uVNTR | Anxiety sensitivity | Early life experiences | Germany |
| Fraley C, 2013 | OXTR rs53576 + rs2254298 / DRD2 rs1800497 / HTR2A rs6313 / OPRM1 rs1799971 | Attachment | Care giving environment | USA |
| Kretschmer T, 2013 | DRD4 7R+ | Delinquency | Negative + positive peer experiences | Netherlands |
| Willoughby MT, 2013 | BDNFVal66Met | ODD + callous–unemotional behaviors | Observed parental behavior | USA |
| Aas M, 2013 | BDNFval66met | Cognitive and brain abnormalities in psychoses | Child trauma | Norway |
| Agnafors S, 2013 | 5HTTLPR/BDNF Val66Met A/G SNP rs6265 | Behavior problems | Early mother depression + child trauma | Sweden |
| Bradley B, 2013 | OXTRrs53577 | Adult resilient coping and positive affect | Child family environment | USA |
| Carballedo A, 2013 | BDNFVal66Met | Hippocampal volume | Early adverse life events | Ireland/Germany |
| Clark R, 2013 | COMT Val158Met | Ptsd | Traumatic load | USA |
| Comasco E, 2013 | 5HTTLPR/BDNFVal66Met | Depression | Child adversity | Sweden |
| Cruz-Fuentes CS, 2013 | BDNFVal66Met /5HTTLPR | Depression | Child adversities | Mexico |
| Hemmings SMJ, 2013 | BDNFVal66Met | OCD | Child trauma | South Africa |
| Jiang R, 2013 | BDNFVal66Met | Depressive symptoms | Adult chronic stress | USA |
| Jonas W, 2013 | OXT rs2740210; rs4813627 / OXTR rs237885 | Depression + breastfeeding duration | Child trauma | Canada |
| Lavigne JV, 2013 | 5HTTLPR/ DRD4 7R+ /MAOA-uVNTR | Oppositional defiant disorder, anxiety, depression | Contextual + parenting risk factors | USA |
| Shinozaki G, 2013 | 5HTTLPR rs25531 | Suicide attempt | Child abuse | USA |
| Melas PA, 2013 | MAOA-uVNTR | DNA methylation of MAOA and NR3C1 | Early-life adversity | Sweden |
| Hill j, 2013 | MAOA-uVNTR | Infant negative emotionality | Early life events during pregnancy | UK |
| van Roekel E, 2013 | OXTR rs53576 | Loneliness | Parental support | Netherlands |
| Brown GW, 2014 | BDNF Val66Met | Depression | Recent life events and child maltreatment | UK |
| Bogdan R, 2014 | 5HTTLPRrs25531 | Depression | Stressful life events | USA |
| Klauke B, 2014 | NPSR rs324981 A/T | Anxiety sensitivity | Child + recent adverse events | Germany |
| Vinberg M, 2014 | 5HTTLPR | Psychiatric disorder | Life stressful events | Denmark |
| Juhasz G, 2014 | Haplotypes GALR1, GALR2, GALR3 | Depression | Child adversity + recent negative life events | UK/Hungary |
| Conway CC | 5HTTLPRrs25531 | Transdiagnostic emotional outcomes | Ideographically assessed daily life stress | USA |
| Zohsel K | DRD4 7R+ | Antisocial outcomes | Mothers’ prenatal stress | Germany |
| Rabl U, 2014 | COMTVal158Met /BDNF Val66Met /5HTTLPR rs25531 | Hippocampal volume | Early and recent Life events | Austria/Germany |
| Walsh K, 2014 | 5HTTLPR rs25531 | PTSD | Child emotional abuse | USA |
| Vogel S, 2014 | NR3C2mineralocorticoid receptor gene | Negative memory bias | Life adversity | Netherlands |
| Dunn EC, 2014 | BDNF rs6265/ CACNA1C rs1006737 / CRHR1 rs12944712/ FKBP5 rs1360780, rs9296158, rs9470080 / OXTR rs53576, rs2254298/ RGS2 rs4606/ 5HTTLPR rs25531 | PTSD | Hurricane exposure + low social support | USA |
| McCarthy-Jones S, 2014 | FOXP2rs1456031, rs2396753, rs2253478 | Auditory verbal hallucinations in EQZ | Parental child abuse | Australia |
| Buchmann AF, 2014 | FKBP5rs1360780 | Cortisol stress response | Child adversity | Germany |
| Cents RAM, 2014 | 5HTTLPR | Child social fearfulness | Maternal sensitivity | Netherlands |
| Cheon B, 2014 | 5HTTLPR | Intergroup bias and prejudice | Out-group threat | USA |
| Davies PT, 2014 | 5HTTLPR rs25531 | Child externalizing symptoms | Maternal unresponsiveness | USA |
| Farbiash T, 2014 | DRD4 7R+ | Aggression | ADHD parental symptoms | Israel |
| Kim JM, 2014 | 5HTTLPR | Suicidal ideation | Life events and social support deficits | Korea |
| Kretschmer T, 2014 | BDNF val66met | Own Aggression | Peer antisocial behavior | UK |
| Richards JS, 2014 | DAT1 9R/10R / DRD4 7R+ / 5HTTLPR | Prosocial and antisocial behaviour in ADHD and their siblings | Maternal expressed emotion | Netherlands |
| Slof-Op’t Landt MCT, 2014 | MR rs5522 / GR rs41423247 | Perfectionism levels in patients eating disorder | Life events | Netherlands |
| Smearman EL, 2014 | OXTRrs53576 | Antisocial behaviors | Social stress | Australia |
| Thompson SM, 2014 | OXTRrs53576 | Depressive symptoms | Early maternal depression | Australia |
| van Winkel M, 2014 | BDNFVal66Met | Negative or positive effect in response to daily events | Social stress sensivity in daily life | Belgium |
| Bortoluzzi A, 2014 | NR3C2MR-2G/C rs2070951 | Serum levels BDNF | Child trauma | Brazil |
| Cicchetti D, 2014 | 5HTTLPR rs25531 /BDNF Val66Met rs4923461 /NET-1014A/G (rs168924) /CRHR1rs110402, rs242924, rs7209436 | Depression + internalizing symptoms | Child maltreatment | USA |
| Dalton ED, 2014 | BDNFval66met /5HTTLPR rs25531 | Depressive symptoms | Family environment | Australia |
| Hernaus D, 2014 | BDNF val66met /FKBP5 rs9296158, rs4713916, rs992105, rs3800373 | Hippocampal Volume + Cognitive Performance | Child trauma | Netherlands |
| Ibarra P, 2014 | BDNF val66me | Psychiatric symptoms | Parenting | Spain |
| Myers AJ, 2014 | OXTR (15 SNPs ). | Anxiety, stress, depression | Early life stress | BRAIN RESOURCE INTERNATIONAL DATABASE |
| Nikitopoulos J, 2014 | DRD4 7R+ | Externalizing behavior | Early maternal care | Germany |
| Park S, 2014 | BDNF Val66Met | Externalizing behavior + ADAH | Parenting | Korea |
| Tsuru J, 2014 | BDNF Val66Met | Cortisol response | TSST and electrical stimulation | Japan |
| Zhang K, 2014 | 5HTTLPR | Depression | Stressful life events | China |
| Haberstick BC, 2014 | MAOA-uVNTR | Antisocial behaviors | Child maltreatment | USA |
| Haberstick BC, 2014 | MAOA-uVNTR | Conduct problems + criminal convictions | Child maltreatment | USA |
| Pickles A, 2014 | MAOA-uVNTR | Anger proneness | Maternal sensitivity | UK |
| Vrijsen JN, 2014 | BDNF Val66Met; rs6265/ COMT Val158Met; rs4680 | Biased processing | Child stress | Netherlands |
| Chou KL, 2014 | CRHR1 rs1876831, rs242938 | Loneliness | Infrequent contact with children and low levels of perceived social support from children | UK |
| Hostinar C, 2014 | OXTR rs53576 | Internalizing - externalizing symptoms + perceived social support | Child adolescent maltreatment | USA |
